# Mouse aldehyde-oxidase-4 controls diurnal rhythms, fat deposition and locomotor activity

**DOI:** 10.1038/srep30343

**Published:** 2016-07-26

**Authors:** Mineko Terao, Maria Monica Barzago, Mami Kurosaki, Maddalena Fratelli, Marco Bolis, Andrea Borsotti, Paolo Bigini, Edoardo Micotti, Mirjana Carli, Roberto William Invernizzi, Renzo Bagnati, Alice Passoni, Roberta Pastorelli, Laura Brunelli, Ivan Toschi, Valentina Cesari, Seigo Sanoh, Enrico Garattini

**Affiliations:** 1 Laboratory of Molecular Biology, Department of Molecular Biochemistry and Pharmacology, IRCCS-Istituto di Ricerche Farmacologiche “Mario Negri”, via La Masa 19, 20156, Milano, Italy; 2 Laboratory of Biochemistry and Protein Chemistry, Department of Molecular Biochemistry and Pharmacology, IRCCS-Istituto di Ricerche Farmacologiche “Mario Negri”, via La Masa 19, 20156, Milano, Italy; 3 Laboratory of Neurodegenerative diseases, Department of Neuroscience, IRCCS-Istituto di Ricerche Farmacologiche “Mario Negri”, via La Masa 19, 20156, Milano, Italy; 4 Laboratory of Neurochemistry and Behaviour, Department of Neuroscience, IRCCS-Istituto di Ricerche Farmacologiche “Mario Negri”, via La Masa 19, 20156, Milano, Italy; 5 Analytical Instrumentation Unit, Department of Environmental Health Sciences, IRCCS-Istituto di Ricerche Farmacologiche “Mario Negri”, via La Masa 19, 20156, Milano, Italy; 6 Laboratory of Mass Spectrometry, Department of Environmental Health Sciences; IRCCS-Istituto di Ricerche Farmacologiche “Mario Negri”, via La Masa 19, 20156, Milano, Italy; 7 Department of Agricultural and Environmental Sciences; Università degli Studi di Milano, via Celoria 2, 20133 Milano, Italy; 8 Graduate School of Biochemical and Health Sciences, Hiroshima University, Hiroshima Japan

## Abstract

Aldehyde-oxidase-4 (AOX4) is one of the mouse aldehyde oxidase isoenzymes and its physiological function is unknown. The major source of AOX4 is the Harderian-gland, where the enzyme is characterized by daily rhythmic fluctuations. Deletion of the *Aox4* gene causes perturbations in the expression of the circadian-rhythms gene pathway, as indicated by transcriptomic analysis. AOX4 inactivation alters the diurnal oscillations in the expression of master clock-genes. Similar effects are observed in other organs devoid of AOX4, such as white adipose tissue, liver and hypothalamus indicating a systemic action. While perturbations of clock-genes is sex-independent in the Harderian-gland and hypothalamus, sex influences this trait in liver and white-adipose-tissue which are characterized by the presence of AOX isoforms other than AOX4. In knock-out animals, perturbations in clock-gene expression are accompanied by reduced locomotor activity, resistance to diet induced obesity and to hepatic steatosis. All these effects are observed in female and male animals. Resistance to obesity is due to diminished fat accumulation resulting from increased energy dissipation, as white-adipocytes undergo trans-differentiation towards brown-adipocytes. Metabolomics and enzymatic data indicate that 5-hydroxyindolacetic acid and tryptophan are novel endogenous AOX4 substrates, potentially involved in AOX4 systemic actions.

Aldehyde oxidases (EC 1.2.3.1, AOXs) are molybdo-flavoenzymes characterized by broad substrate specificity^1^,^2^,^3^. AOXs oxidize aldehydes into carboxylic acids and hydroxylate aromatic heterocycles. The active form of AOXs is a 300 kDa homodimer^4^,^5^,^6^. The number of mammalian AOX isoenzymes varies according to the species considered^7^. Humans are characterized by a single enzyme, AOX1, while rodents synthesize four isoenzymes, AOX1, AOX2 (previously AOX3L1), AOX3 and AOX4. In mice, AOXs are encoded by distinct genes, clustering on chromosome 1^7^. In humans, the vestiges of the mouse *Aox* gene-cluster are identifiable on chromosome 2, where *AOX1* lays next to the mouse *Aox3* and *Aox2* orthologs that underwent a process of pseudogenization^7^.

The physiological function and substrates of mammalian AOXs are obscure, although human and mouse liver AOX isoenzymes play a role in phase I metabolism of xenobiotics^8^,^9^,^10^. *In vitro* studies suggest that mouse AOX isoenzymes have overlapping substrate specificities^11^,^12^. Among the substrates of potential physiological relevance, *all-trans* retinaldehyde (RAL), the metabolic intermediate of vitamin A, is oxidized by mouse AOXs into *all-trans* retinoic acid (ATRA)^13^,^14^,^15^. The four mouse AOXs are proposed to exert distinct functions given their tissue-specific pattern of expression. AOX1 is relatively ubiquitous, although high levels of the corresponding mRNA and protein are found in liver and lung^5^,^12^,^16^. The expression profiles of AOX3 and AOX1 mRNAs as well as proteins are largely overlapping^5^. High levels of AOX2 are observed only in the Bowman’s glands of the nasal cavity^13^. AOX4 is expressed in the tongue, oesophagus and epidermis^12^,^14^. Nevertheless, the Harderian-gland (*HG*) is by far the richest source of AOX4^14^ where the enzyme represents 2% of all cytosolic proteins. *HG* is a large exocrine gland of the intra-orbital cavity^17^ and it is conserved in most vertebrates with the exception of primates. *HG* physiological function is incompletely defined, although it is involved in the control of the diurnal cycle, as it synthesizes large amounts of the photodynamic compounds, protoporphyrin IX^18^ and melatonin^19^. *HG* is also involved in eye and fur coat lubrication *via* production of a lipid-rich secretion^17^. Specific physiological processes in the rodent *HG*, such as lipid and protein metabolism, show rhythmic fluctuations^20^,^21^,^22^.

The present work provides novel insights into the physiological function of AOX4 which result from an integration of the phenotypic, transcriptomic and metabolomic data obtained in *Aox4* knock-out (*Aox4*^*−/−*^) mice^14^.

## Results

### Perturbations of periodical clock-gene expression in HG of *Aox4* ^−/−^ mice

**Figure 1:**
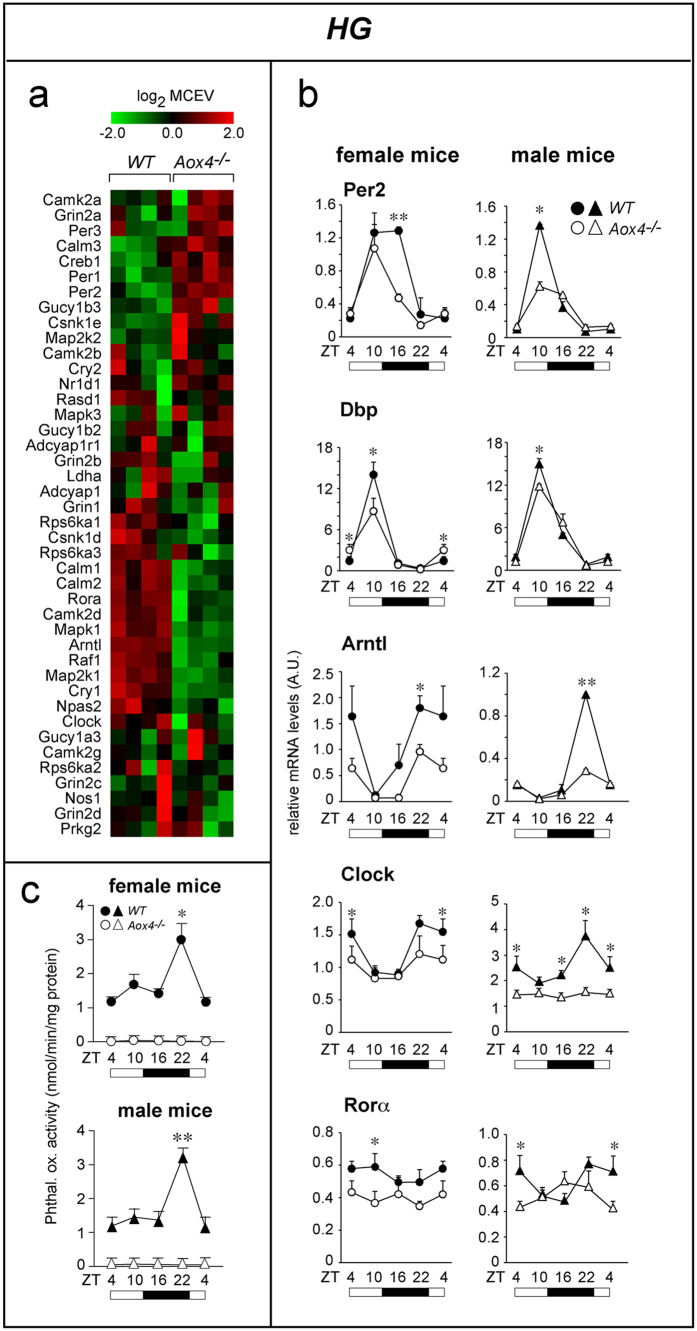
Circadian-rhythm genes in HG of *Aox4*^−/−^ and WT mice. (**a**) The heat maps indicate the expression levels of the mRNAs belonging to the METACORE “Circadian-rhythms” pathway in *HG*. Data are shown as the log_2_ of the median centred expression values (MCEV). (**b**) The linear graphs show the levels of the indicated clock mRNAs measured by PCR at the indicated zeitgebers (*ZT*). Values are the mean ± SE of 4 mice. (**c**) The two graphs show the diurnal oscillations of AOX4 enzymatic activity in *HG* cytosolic extracts of female and male *Aox4*^*−/−*^ and *WT* mice using phthalazine as substrate. All the enzymatic assays were performed in the absence of NAD as a cofactor. In *Aox4*^*−/−*^ mice, the levels of phthalazine oxidizing activity are below detection. The first points (ZT = 4) are repeated at the end of the graph to better represent the diurnal oscillations. Values are the mean ± SE of 4 mice. White boxes = light-phase; Black boxes = dark-phase. Significantly different relative to the *Aox4*^*−/−*^ corresponding value; *(Student’s t-test, p < 0.05); **(Student’s t-test, p < 0.01) *WT vs Aox4*^*−/−*^ mice.

The gene-expression profiles of *HG* in female AOX4 knock-out (*Aox4*^*−/−*^)^14^ and wild-type (*WT*) mice were compared. *Aox4*-deletion is associated with differential expression of 759 probes (log_2_ fold-change ± 0.5; p-value < 0.005), most of which are down-regulated (658 probes), as indicated in Suppl. Table 1. Pathway analysis (Suppl. Table 1) indicates enrichment of gene networks controlling the immune response such as “Immune response_CD16 signalling in NK cells” (FDR = 2.3 e^−4^). Other enriched gene-sets, such as “Regulation of lipid metabolism” (FDR = 6.0 e^−4^), “Insulin, IGF-1 and TNF-alpha in brown adipocyte differentiation” (FDR = 1.4 e^−2^) control lipid homeostasis. One of the top gene signatures significantly enriched in *Aox4*^*−/−*^ mice is relevant for the purported role of *HG* in the light/dark cycle^22^ (Fig. 1a and Suppl. Fig. S1a). This METACORE signature (“Circadian-rhythm”) ranks 3^rd^ among the top perturbed ones (FDR = 2.9 e^−4^). AOX4 deletion alters the expression of circadian rhythm genes, like *Arntl* (also known as *Bmal1*) *Per1*, *Per2*, *Clock* or *Cry1*, and other clock-genes, like *Dbp*, which are not present in the METACORE “Circadian-rhythm” pathway (Suppl. Table 1). The expression profiles of three major clock-genes, *Per2*, *Dbp* and *Arntl*, were validated by PCR (Suppl. Fig. S1b). It must be noticed that the microarray data were obtained at a single time point during the diurnal cycle, between zeitgebers (*ZT*) 1–4 (*ZT*0 = 7 a.m., lights-on; *ZT*12 = 7 p.m., lights-off), as the observed perturbations on clock genes were unexpected.

Clock-genes are characterized by diurnal and cyclic variations in their expression. To get insights into the dynamic effects exerted by *Aox4*-deletion on selected clock-genes, we evaluated the expression levels of *Per2*, *Dbp*, *Arntl*, *Clock*, *RORα* and *Rev-erbβ* every six hours up to 24 hours in an independent set of experiments (Fig. 1b and Suppl. Fig. S2). As expected, these mRNAs show cyclic profiles of expression with peaks at different *ZT*s (*Per2* = 10/16; *Dbp* = 10; *Arntl* = 22; *Clock* = 22; *RORα* = 4/10; *Rev-erbβ* = 10) in female *WT* mice. *Aox4*-deletion causes an overall reduction in the oscillation amplitude of *Per2*, *Dbp*, *Arntl*, *Clock* and *RORα* mRNAs, with minor shifts in the oscillation phase (Fig. 1b). As for the oscillatory pattern of the clock gene *Rev-erbβ* (*Nr1d2*), whose expression was not altered on the basis of microarray data, it is virtually identical in *Aox4*^*−/−*^ and *WT* mice (Suppl. Fig. S2).

Although *Aox4* expression in *HG* is similar in male and female animals, there is a restricted time window in which the enzyme shows sexual dimorphism^14^. As all the above studies were conducted in females, we looked for potential sex differences in *Aox4*^*−/−*^ perturbations of clock-genes (Fig. 1b and Suppl. Fig. S2). The diurnal expression profiles of each clock mRNA can vary in *WT* female and male animals. However, relative to *WT*, *Aox4*^*−/−*^*HG* of both female and male mice present with reductions in the amplitude of *Per2*, *Dbp*, *Arntl*, *Clock* and *RORα* diurnal oscillations, indicating that regulation of these genes by AOX4 is not influenced by sex. Interestingly, the diurnal oscillatory patterns of *Arntl* and *Clock* in *Aox4*^*−/−*^and *WT* mice of both sexes is remarkably similar, consistent with the observation that the two transcription factors act as heterodimers^23^.

AOX4 may control clock-gene periodical expression *via* a substrate/product of the enzyme. Thus, we evaluated whether *HG* AOX4 enzymatic activity is also characterized by a daily oscillatory pattern and whether this is not influenced by sex. We measured AOX4-dependent oxidizing activity of phthalazine, a specific substrate, in female and male *WT* animals at the same *ZTs* considered for clock genes. Indeed, *HG* AOX4 enzyme activity varies with the diurnal cycle, being low during the light-phase and high at the end of the dark-phase (Fig. 1C). The overall levels and the light/dark profiles of AOX4-dependent phthalazine-oxidizing activity are very similar in male and female *WT* mice. These data support the idea that disruption of the diurnal variations in the local levels of one or more molecules directly or indirectly controlled by AOX4 is at the basis of the perturbations in clock-gene expression observed in *Aox4*^*−/−*^*HG*.

### Potential novel AOX4 substrates relevant for the control of circadian-rhythm genes in HG

To identify potential mediators of the AOX4-dependent effects on clock gene expression, we initially focused on two photo-sensors, protoporphyrin-IX and melatonin, which are abundant in *HG*s^18^. Protoporphyrin IX levels were evaluated in female *WT* and *Aox4*^*−/−*^ animals at four *ZT*s (Suppl. Fig. S3a). The porphyrin content is higher in knock-out mice at three of the four *ZT*s, suggesting that AOX4 modulates the levels of protoporphyrin IX in *HG. Aox4*^*−/−*^ mice are stabilized on a *C57BL/6J* background, which has a melatonin deficit due to the absence of hydroxyindole O-methyltransferase (HIOMT; EC 2.1.1.4)^24^. Consistent with this, both *WT* and *Aox4*^*−/−*^animals are characterized by serum melatonin levels which are below the detection limit of the mass-spectrometry method used to determine the hormone in HIOMT-proficient *C3H/HeJ* mice (Suppl. Fig. S3b). Thus, while protoporphyrin IX may be involved in AOX4-dependent control of clock-genes, melatonin is unlikely to mediate the effects on clock-gene expression observed in *Aox4*^*−/−*^mice.

To identify other endogenous mediators, we performed untargeted differential metabolomic studies in the *HG* of female mice (Suppl. Fig. S4). We identified 25 metabolites distinguishing *Aox4*^*−/−*^ from *WT* animals (Suppl. Table S2). Among them, tryptophan and 5-hydroxy-indolacetic acid (5HIAA) drew our attention, as they are part of the serotonin/melatonin biosynthetic pathway (Suppl. Fig. S5), which controls circadian-rhythms. Tryptophan is more abundant in *Aox4*^*−/−*^ than *WT* mice, while 5HIAA is measurable only in *Aox4*^*−/−*^ mice. The chemical structures of 5HIAA and tryptophan suggest that they are potential AOX substrates. To support this hypothesis, purified *HG AOX4* was incubated with tryptophan, 5-HIAA and serotonin (Suppl. Fig. S6a). AOX4 recognizes tryptophan and 5-HIAA as substrates, while the enzyme does not metabolize the structural analogue, serotonin. 5HIAA and tryptophan are metabolized also by purified liver AOX3. Mass-spectrometric analysis of the reaction mixtures indicate that AOX4 and AOX3 oxidize 5HIAA and tryptophan to mono-hydroxylated products (Suppl. Fig. S6b,c). As for tryptophan, incubation of the compound with both AOX4 and AOX3 results in the same three mono-hydroxylated products. The mass fragmentation profiles of the 3 peaks are distinct from that of 5-OH-tryptophan, the product of tryptophan-hydroxylases. These data suggest that 5HIAA and tryptophan or derived metabolites represent direct mediators of the AOX4-dependent effects on clock-genes.

### Sex influence on clock-gene perturbations in tissues other than HG

The action of AOX4 on clock-genes may be part of systemic effects involving tissues other than *HG*. Thus, we compared the gene-expression profiles of visceral white-adipose-tissue (*WADT*) and liver of female *Aox4*^*−/−*^and *WT* mice. *WADT* and liver do not express AOX4, although they contain significant amounts of other AOXs, *i.e* AOX1 in *WADT* and AOX3 plus very low levels of AOX1 in liver^15^. In addition *WADT* and liver are representative of tissues having a major role in energy metabolism and lipid homeostasis, two processes potentially influenced by circadian rhythms^25^,^26^,^27^,^28^.

**Figure 2:**
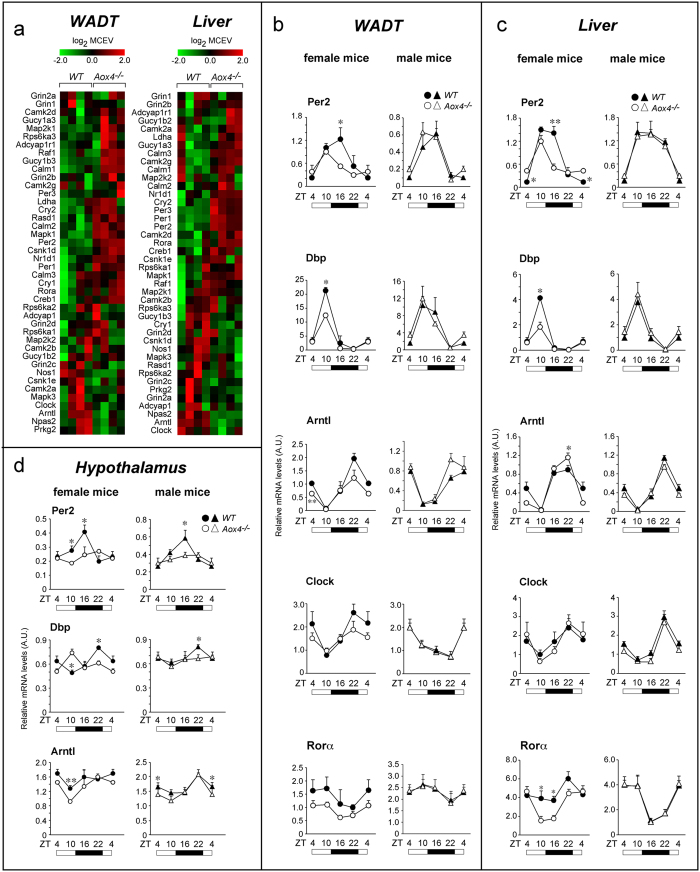
Circadian-rhythm genes in *Aox4*^*−/−*^ WADT, liver and hypothalamus of *Aox4*^*−/−*^ and WT mice. (**a**) The heat maps indicate the expression levels of the mRNAs belonging to the METACORE “Circadian-rhythms” pathway in *WADT* and liver, as indicated. Data are shown as the log_2_ of the median centered expression values (MCEV). (**b–d**) The linear graphs show the levels of the indicated clock mRNAs measured by PCR at the indicated zeitgebers (*ZT*) in *WADT*, liver and hypothalamus. The first points (ZT = 4) are repeated at the end of the graph to better represent the diurnal oscillations. White boxes = light-phase; Black boxes = dark-phase. Values are the mean ± SE of 4 mice. *(Student’s t-test, p < 0.05); **(Student’s t-test, p < 0.01) *WT vs Aox4*^*−/−*^ mice.

A large number of genes is modulated in *Aox4*^*−/−*^*WADT* and liver selectively (Suppl. Table S1). The number of differentially regulated genes (log_2_ fold-change ±0.5; p-value < 0.005) is 601 in *WADT* and 962 in liver. In *WADT* and liver, pathway enrichment analysis indicates that the genes are organized in 48 and 20 overlapping networks (FDR < 0.05), respectively. Similar to what is observed in *HG*, “Circadian-rhythm” is ranking among the top enriched pathways in *WADT* and liver of *Aox4*^*−/−*^animals (Suppl. Table S1). The differential expression profiles of the individual genes belonging to the “Circadian-rhythm” pathway in *WADT* and liver of female *Aox4*^*−/−*^mice are shown in Fig. 2a. In *WADT* and liver of female *Aox4*^*−/−*^mice, the expression patterns of *Per2*, *Dbp* and *Arntl* clock-genes are similar to those observed in *HG* (Fig. 2a and Suppl. Fig. S1a). The microarray results obtained in *WADT* and liver were validated by real-time PCR (Suppl. Fig. S1c,d).

Evaluation of the daily oscillatory patterns of *Per2, Dbp* and *Arntl* in female *WADT* and liver confirms what was observed in *HG*, as *Aox4*-deletion reduces the amplitude of the diurnal oscillations of these genes (Fig. 2b,c). A similar trend is observed for *Clock* and *Rorα*, although the results reach statistical significance only for *Rorα* in liver. No difference in the diurnal oscillations of *Rev-erbβ* is observed between *Aox4*^*−/−*^and *WT* animals in the two tissues (Suppl. Fig. S2). Interestingly, male *Aox4*^*−/−*^mice do not show the reduction in the oscillation amplitudes of *Per2*, *Dbp*, *Arntl*, *Clock* and *Rorα* observed in the female WADT and liver (Fig. 2b,c). We also explored whether AOX4 modulates *Per2, Dbp* and *Arntl* expression in the hypothalamus, which is devoid of any AOX isoenzyme and contains the supra-chiasmatic nucleus, *i.e.* the central controller of circadian-rhythms (Fig. 2d). As observed in *HG*, *WADT* and liver, both female and male *Aox4*^*−/−*^ mice show a reduction in the daily oscillation amplitude of these mRNAs in the hypothalamus too. These data indicate that sex exerts a tissue-specific influence on the perturbations in the expression of specific clock genes afforded by *Aox4* deletion.

### Contribution of AOX3 to sex-dependent clock-gene regulation in liver

**Figure 3:**
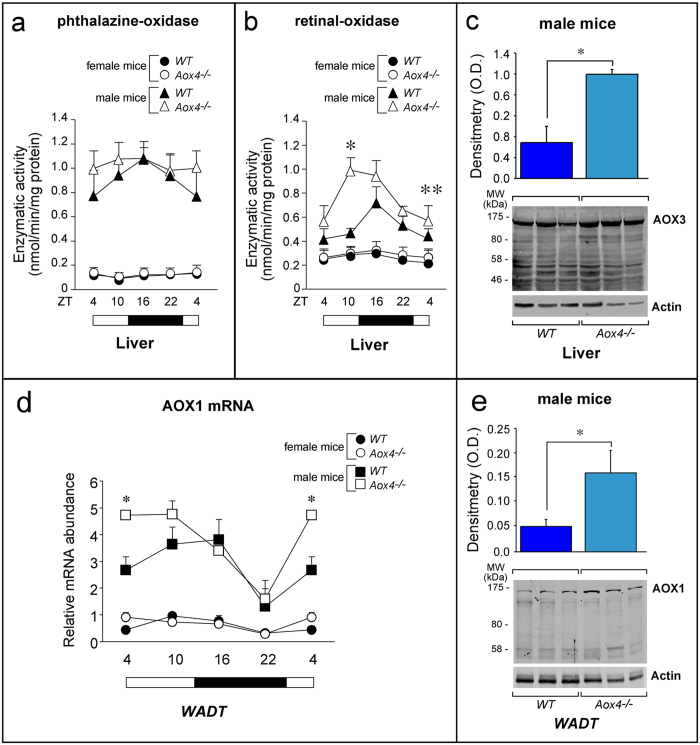
Expression of AOX3 and AOX1 mRNAs and proteins in liver and WADT. (**a**,**b**) The two graphs show the diurnal oscillations of AOX4 enzymatic activity in liver cytosolic extracts (**a**, substrate = phthalazine; **b**, substrate = retinal). All the enzymatic assays were performed in the absence of NAD as a cofactor. Values are the mean ± SE of 4 mice. White boxes = light-phase; Black boxes = dark-phase. Significantly different relative to the *Aox4*^*−/−*^ corresponding value; *(Student’s t-test, p < 0.05); **(Student’s t-test, p < 0.01). (**c**) Western-blot analysis of the liver AOX3 protein (ZT = 4) in *Aox4*^*−/−*^ and *WT* animals is shown. The densitometric quantitation of the Western blot results is illustrated by the bar graphs following normalization for the actin signal. Each lane represents a single animal. The results are expressed as the mean ± S.E. *Significantly different (Student’s t-test, p < 0.05). (**d**) The line graph illustrates the expression levels of the AOX1 mRNA in the *WADT* of the indicated animals at different zeitgebers, as assessed with a specific PCR assay. White boxes = light-phase; Black boxes = dark-phase. Values are the mean + S.E. of 4 animals. The first points (ZT = 4) are repeated at the end of the graph to better represent the diurnal oscillations. (**e**) Western-blot analysis of the *WADT* AOX1 protein (ZT = 4) in *Aox4*^*−/−*^ and *WT* animals is shown. The densitometric quantitation of the Western blot results is illustrated by the bar graphs following normalization for the actin signal. Each lane represents a single animal. The results are expressed as the mean ± S.E. *Significantly different (Student’s t-test, p < 0.05).

AOX3, AOX1 and AOX4, act on largely overlapping sets of substrates^14^,^15^. In addition, hepatic AOX3 is sex and testosterone dependent, with males expressing much larger amounts of the protein than females^16^. Thus, we evaluated whether the sex-dependent differences in the clock-gene perturbations observed in liver and *WADT* of *Aox4*^*−/−*^ mice may be associated with altered amounts of hepatic AOX3 and adipocyte AOX1^15^. In liver, low and similar levels of phthalazine-oxidase activity are detectable in *WT* and *Aox4*^*−/−*^females throughout the diurnal cycle (Fig. 3a). *WT* males show at least ten-fold higher levels of phthalazine-oxidase activity than females. Surprisingly, the amounts of this enzymatic activity are even larger in male *Aox4*^*−/−*^ mice. This male-specific difference in *Aox4*^*−/−*^ animals is more evident if the production of ATRA from RAL is measured (Fig. 3b). Hence, male *Aox4*^*−/−*^mice seem to activate a compensatory response to *HG* AOX4 deficiency by increasing hepatic AOX3 protein in liver, as indicated by the Western blot data (Fig. 3c). A similar compensatory increase of AOX enzymatic activity is likely to occur in male *Aox4*^*−/−*^*WADT* due to AOX1 mRNA and protein up-regulation (Fig. 3d,e). Induction of liver AOX3 and *WADT* AOX1 are likely to influence the local production of substrate(s)/metabolite(s) common to AOX3, AOX1 and AOX4. This may explain the lack of effects on *Per2*, *Dbp, Arntl Clock* and *Rorα* diurnal oscillations observed in the two tissues of male animals.

### Female and male *Aox4* ^−/−^ animals present with reduced locomotor activity

**Figure 4:**
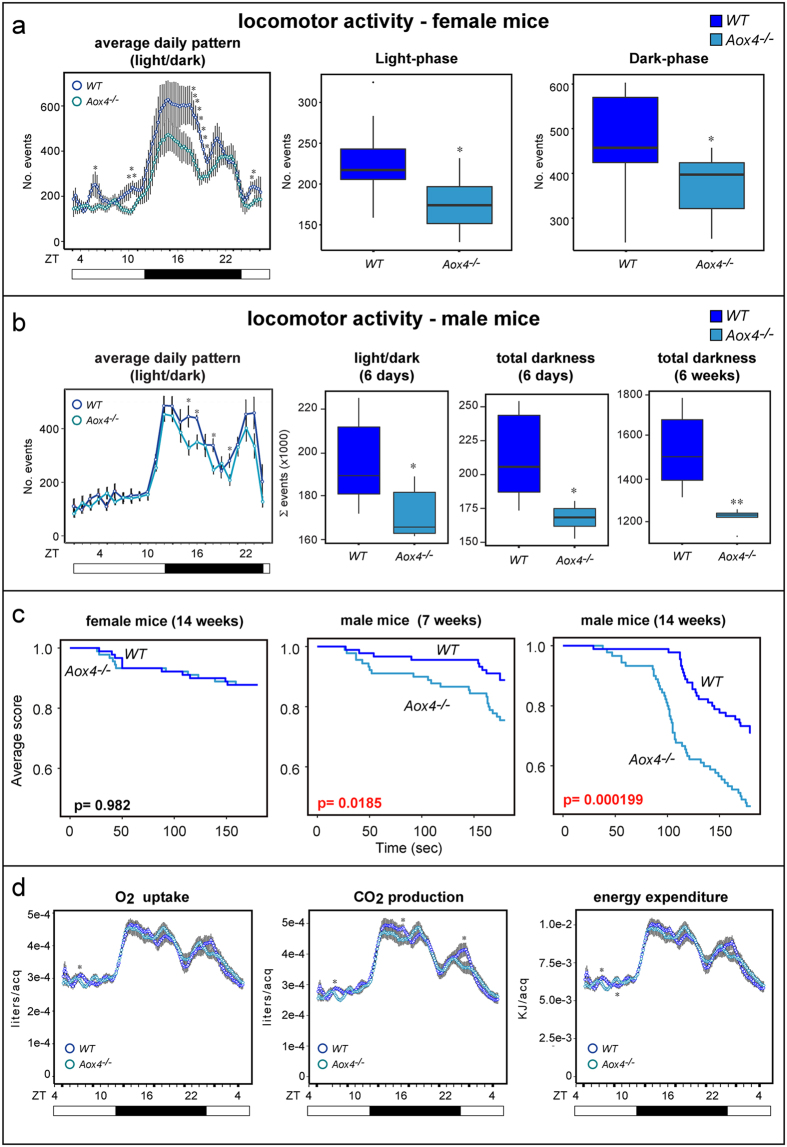
Locomotor activity, muscle strength and energy expenditure in *Aox4*^*−/−*^ and WT mice. (**a,b**) Left: The locomotor activity of female (**a**) and male (**b**) *Aox4*^*−/−*^ and *WT* mice was measured in infrared-sensor monitored cages throughout 24 hours for 15 days (mean ± SD of 6 mice). *ZT* = zeitgeber. White boxes = light-phase; Black boxes = dark-phase. Middle and Right: The box plots illustrate the overall locomotor activity observed during day and night time as indicated. Values are the median ± SD of 6 mice. (**c**) Female and male animals of the indicated ages were subjected to the hanging wire test. In the case of males, 9 *WT* and an equivalent number of *Aox4*^*−/−*^ mice were used. As for females, 8 *WT* and 9 *Aox4*^*−/−*^ mice were used. The Average Score was calculated as described in Materials and Methods. The higher is the Average Score, the lower is the number of times the animals fall from the hanging wire. (**d**) Female mice were subjected to indirect calorimetry experiments. Values are the mean ± SD of 20 mice. ACQ = acquisition. Significantly different relative to the *Aox4*^*−/−*^ corresponding value; *(Student’s t-test, p < 0.05); **(Student’s t-test, p < 0.01).

Clock-gene modifications may affect animal behavior, which led us to compare locomotor activity in female *Aox4*^*−/−*^and *WT* mice across the circadian cycle. During the dark-phase, two activity peaks are evident in *WT* mice (*ZT* = 13–17,20). The height of the two peaks is reduced in *Aox4*^*−/−*^ animals (Fig. 4a). During the light-phase, three small peaks, disappearing in *Aox4*^*−/−*^ animals, are evident in *WT* mice (*ZT* = 1,6,10). Regardless of the light/dark phase, female *Aox4*^*−/−*^mice show an overall decrease in locomotor activity relative to *WT* animals. Although minor sex-dependent differences in the daily motile pattern of *WT* and *Aox4*^*−/−*^mice are observed, the overall reduction in locomotor activity is confirmed in *Aox4*^*−/−*^males (Fig. 4b). In the case of male *Aox4*^*−/−*^ and *WT* mice, only cumulative data are presented, although a significant reduction in locomotor activity is observed both during the light- and dark-phase. The motile behaviour of *Aox4*^*−/−*^and *WT* animals was also compared in male mice adapted to constant darkness for two different periods of time (6 days and 6 weeks). In these conditions, the reduction in locomotor activity of *Aox4*^*−/−*^mice is maintained and become more significant with time (Fig. 4b). This indicates that AOX4 controls locomotor activity *via* mechanisms independent of light and sex.

Muscle strength deficits may contribute to decreased locomotor activity of *Aox4*^*−/−*^mice. Hence, we subjected animals to hanging wire tests, which measure muscle strength (Fig. 4c). Mature (14–15 weeks) *Aox4*^*−/−*^females show equivalent hanging times as *WT* animals, while a mild deficit is present in young (7 weeks) *Aox4*^*−/−*^males. This deficit becomes more evident in mature (14 weeks old) animals, and it is not accompanied by weight loss of the gastrocnemius, soleus and tibial muscles. It is interesting that the diameter of the tibial muscle fibers (see Materials and Methods) in *Aox4*^*−/−*^male mice is lower than in *WT* animals (Mean ± SE: *Aox4*^*−/−*^ = 37.5 ± 0.3, *WT* = 43.7 ± 0.3; Student’s t-test p = 6.2 E^−35^). A similar difference in fiber size is not evident in the female counterparts (Mean ± SE: *Aox4*^*−/−*^ = 38.4 ± 0.3, *WT* = 38.8 ± 0.1; Student’s t-test p = 0.38). These last results may partially explain why muscle weakness is specifically observed in male animals. In conclusion, the deficit in locomotor activity observed in both female and male *Aox4*^*−/−*^ animals is unlikely to be due to muscular atrophy.

Decreased *Aox4*^*−/−*^locomotor activity may cause alterations in energy homeostasis. Consequently, we subjected female animals to indirect calorimetry. In *WT* and *Aox4*^*−/−*^ animals, the daily profiles of locomotor activity and energy expenditure, calculated from oxygen consumption and carbonic anhydride production, are similar (Fig. 4d). Overall, energy expenditure is also similar in *WT* and *Aox4*^*−/−*^ mice, except for a small, but significant increase of this parameter in *Aox4*^*−/−*^ mice at *ZT*9.

### *Aox4* ^−/−^ mice have a lean phenotype due to low feed efficiency

Circadian clocks regulate body weight *via* control of fat deposition^29^,^30^. Moreover, *HG* metobolomics suggest a role for AOX4 in lipid homeostasis (Suppl. Table S2). In fact, many metabolites differentially regulated in *Aox4*^*−/−*^*HG* are lipids, including cholesterol, phospholipids (lysophosphatidylcholines; phosphatidylethanolamines; lysophosphatidylethanolamine), linoleic acid and dihomo-γ-linolenoyl-ethanolamide. Alterations in HG lipid metabolism may be consequent to more general effects on cell energy balance, as suggested by the observation that panthotenate is measurable only in *Aox4*^*−/−*^ mice. The increase in N(6)-(1,2 dicarboxyethyl) AMP, a fumarate precursor, may reflect stimulation of the citrate cycle. High panthotenate levels in *Aox4*^*−/−*^ animals may be due to augmented energy wasting *via* enhanced mitochondrial activity. In *Aox4*^*−/−*^ mice, isobutyryl-L-carnitine depletion may also be consistent with increased mitochondrial oxidative activity, as the compound is the product of acyl-CoA dehydrogenases, which are mitochondrial enzymes involved in fatty acid oxidation. Indeed, crude mitochondrial fractions of *HG* from female *Aox4*^*−/−*^ mice contain significantly larger amounts of citrate synthase and ATP synthase (complex-V) than the *WT* counterparts and a trend towards higher levels of complex-I to complex-IV (Suppl. Fig. S7), supporting an increase in the number and activity of these organelles. Finally, process analysis of the *HG* microarray data (Suppl. Table S1) indicates the AOX4-deficiency affects lipid metabolism.

**Figure 5:**
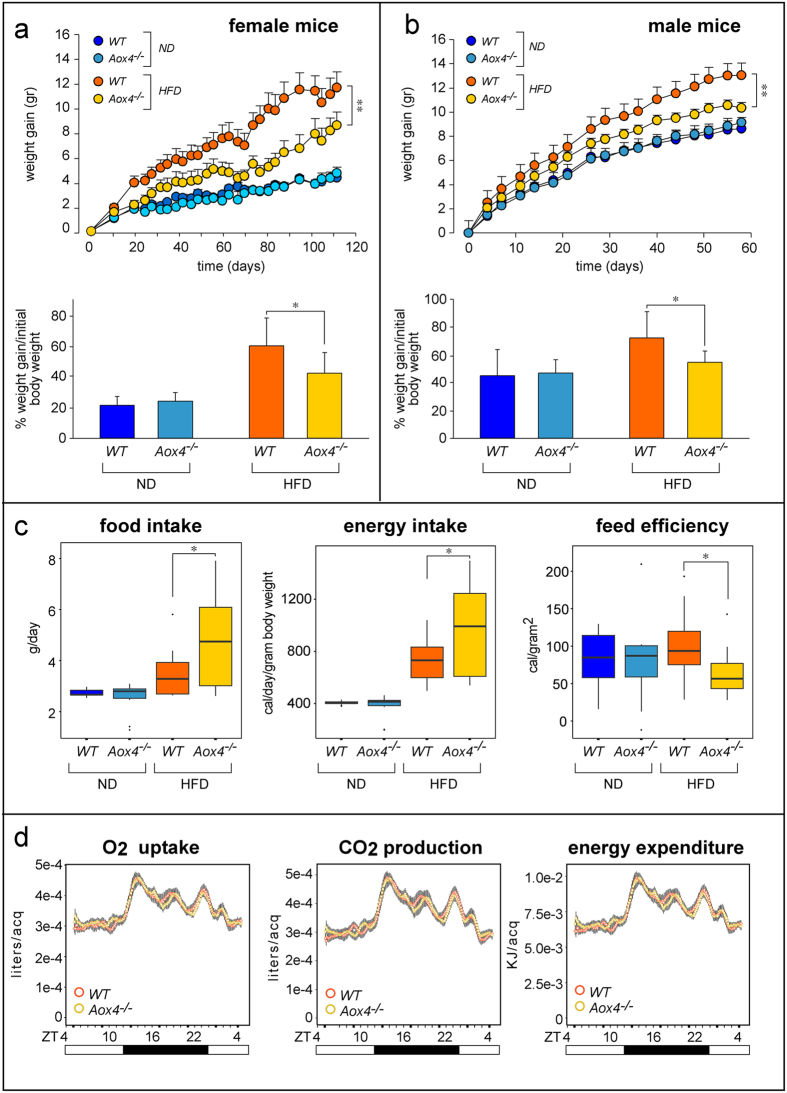
Lean phenotype of *Aox4*^*−/−*^ mice. Female or male *WT* and *Aox4*^*−/−*^ mice were subjected to normal (*ND*) and high fat (*HFD*) diet for 110 or 60 days. (**a**,**b**) Upper: The panels show the weight gain curves of female and male *WT* and *Aox4*^*−/−*^ mice. Values are the mean ± SD of 10 mice. **Significantly different (Student’s t-test, p < 0.01). Lower: The graphs indicate the daily weight gain normalized for the initial weight of *WT* and *Aox4*^*−/−*^ mice which was obtained from the growth curves. (**c**) The box plots show food intake, energy intake and feed efficiency (Median ± SD of 10 mice). *(Student’s t-test, p < 0.05) and **(Student’s t-test, p < 0.01), significantly different relative to the *Aox4*^*−/−*^ corresponding value. (**d**) Female animals fed *HFD* for 1 month were subjected to indirect calorimetry experiments. Each panel illustrates the daily profile measured for the indicated parameter. Each value is the mean ± SD of 16 distinct animals. acq = acquisition. ZT = zeitgeber. White boxes = light-phase; Black boxes = dark-phase.

Given this background, we evaluated whether *Aox4*-deletion affects body weight in female mice fed standard (*ND*) or high-fat (*HFD*) diets (Fig. 5a). *ND* fed *Aox4*^*−/−*^ mice show similar body weight-gain as *WT* mice. *HFD* causes a much lower increase in the weight of *Aox4*^*−/−*^ than *WT* mice. The lean phenotype of *Aox4*^*−/−*^ animals is further supported by the weight-gain values after normalization for the initial body weight. These effects are confirmed in male animals (Fig. 5b). Hence, both female and male *Aox4*^*−/−*^ mice show equal resistance to diet-induced increase in body weight, as also indicated by the total body weight curves (Suppl. Fig. S8).

To identify the mechanisms underlying the deficits in weight-gain observed in knock-out mice, we determined food-intake and we calculated energy-intake (food-intake x food-caloric-content) as well as feed-efficiency (daily weight gain/energy intake) in female *Aox4*^*−/−*^ and *WT* animals fed *ND* and *HFD* (Fig. 5c). After normalization for individual body weight, *Aox4*^*−/−*^ and *WT* mice kept on *ND* show no difference in food/energy-intake. These indexes are slightly although significantly higher in *Aox4*^*−/−*^ animals fed *HFD*. Given the reduced weight-gain, *Aox4*^*−/−*^ mice exposed to *HFD* show a lower feed-efficiency than *WT* animals. This is not explained by an overall increase in whole-body energy-expenditure, as indirect calorimetric analysis indicates no difference between *Aox4*^*−/−*^ and *WT* mice fed *HFD* (Fig. 5d), replicating the data observed in basal conditions (Fig. 4d). In addition, we evaluated whether intestinal nutrient absorption or urine production is affected in *Aox4*^*−/−*^ animals kept under standard conditions. No difference in the daily amount of faeces or urine volume is observed in *Aox4*^*−/−*^ and *WT* mice (Suppl. Fig. S9a,b). Furthermore, the content of total lipids in the faeces show no significant difference in *ND*-fed *Aox4*^*−/−*^ and *WT* animals (Suppl. Fig. S9c, left). Upon *HFD*-feeding, an approximate 25% decrease in the faecal lipid content is observed in knock-out relative to *WT* animals, although the results do not reach statistical significance. Finally, qualitative determination of the lipid species present in the faeces by thin layer chromatography indicate that the lipid composition is substantially identical in *Aox4*^*−/−*^ and *WT* mice fed *ND* or *HFD* (Suppl. Fig. S9c, right). Overall, these last data indicate that there is no evidence of deficits in intestinal fat absorption, which may contribute to the lean phenotype of *Aox4*^*−/−*^ animals.

### *Aox4* ^*−/−*^ mice show constitutive deficits in fat deposition

**Figure 6:**
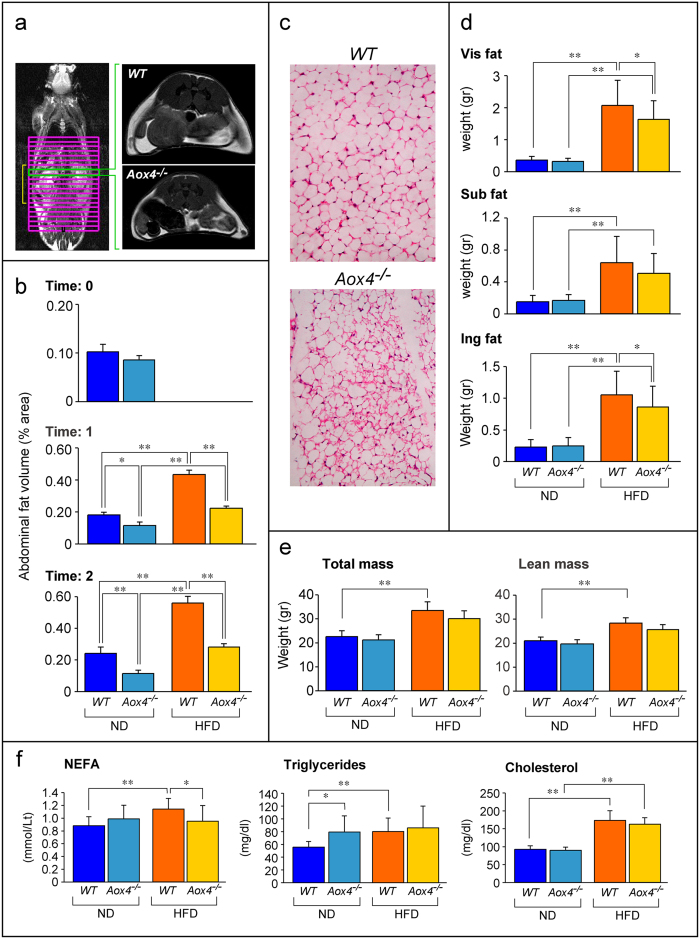
Reduced fat accumulation in *Aox4*^*−/−*^ WADT. (**a**) The panel on the left illustrates a representative whole-body MRI performed on a *WT* mouse. The grid indicates the image sections (yellow square parenthesis) used for the determination of abdominal *WADT* volume. The two right figures show representative images of visceral adipose tissue sections of animals fed *ND*. (**b**) The graphs show the total abdominal fat volume measured by MRI in *Aox4*^*−/−*^ and *WT* animals fed *ND* or *HFD* for 1 month (Time:1) or 2 months (Time:2). Values are the mean ± SD of 6 animals. (**c**) The images show typical microscopic fields (magnification x100) of inguinal *WADT* in mice fed *HFD* for 2 months. (**d**) The graphs illustrate the weight of visceral (Vis), subcutaneous (Sub) and inguinal (Ing) *WADT* in mice subjected to *ND* or *HFD* for 5 weeks. Values are the mean ± SD of 9 mice under *ND* and 16 mice under *HFD*. (**e**) The graphs show the total and lean mass of mice subjected to *ND* or *HFD* for 5 weeks. Values are the mean ± SD of the same number of mice as in (**d**). (**f**) The graphs show the serum levels of NEFA (non-esterified fatty acids), triglycerides and cholesterol measured in *Aox4*^*−/−*^ and *WT* animals subjected to ND and HFD for 2 months. Each value is the mean ± SD of 10 distinct mice. Significantly different: *(Student’s t-test, p < 0.05), **(Student’s t-test, p < 0.01).

We evaluated whether perturbations of lipid homeostasis extend to *WADT*, as altered lipid homeostasis and fat deposition may explain the weight-gain deficits observed in *Aox4*^*−/−*^ mice. The volume of abdominal *WADT* was assessed by Magnetic-Resonance-Imaging (MRI) (Fig. 6a). Two groups of female *Aox4*^*−/−*^and *WT* animals were kept on *ND* and *HFD* (Fig. 6b). Upon *ND* for one month, fat volume is lower in *Aox4*^*−/−*^relative to *WT* mice and the difference becomes more evident after two months. These phenomena are amplified upon *HFD*. The deficit in abdominal fat deposition is supported by *WADT* microscopic analysis in *ND*-fed mice which shows that adipocytes are larger in *WT* than *Aox4*^*−/−*^animals because of a higher lipid content (Fig. 6c). Relative to *ND*-fed mice, the weight of visceral, subcutaneous and inguinal *WADT* is increased in all *HFD*-fed animals. However, the weight increment is lower in *Aox4*^*−/−*^ than *WT* visceral/inguinal fat (Fig. 6d). Similarly, the increase in total and lean body mass of *Aox4*^*−/−*^mice on *HFD* is less pronounced than in *WT* mice (Fig. 6e). These differential effects on lean mass in *Aox4*^*−/−*^and *WT* animals are expected, as obese mice show ectopic fat deposition outside *WADT*^31^.

The fat deposition deficit of *Aox4*^*−/−*^mice is accompanied by perturbations in circulating triglycerides and non-esterified fatty-acids (*NEFA*) (Fig. 6f). Under *ND*, triglyceride serum levels are higher in *Aox4*^*−/−*^than *WT* animals, while *HFD* increases triglyceride circulating levels only in *WT* mice. Similarly, *NEFA* levels are increased solely in *WT* controls exposed to *HFD. Aox4*-deletion has no effect on serum cholesterol regardless of diet.

### WADT of *Aox4* ^−/−^ mice exposed to HFD shows increased energy dissipation

**Figure 7:**
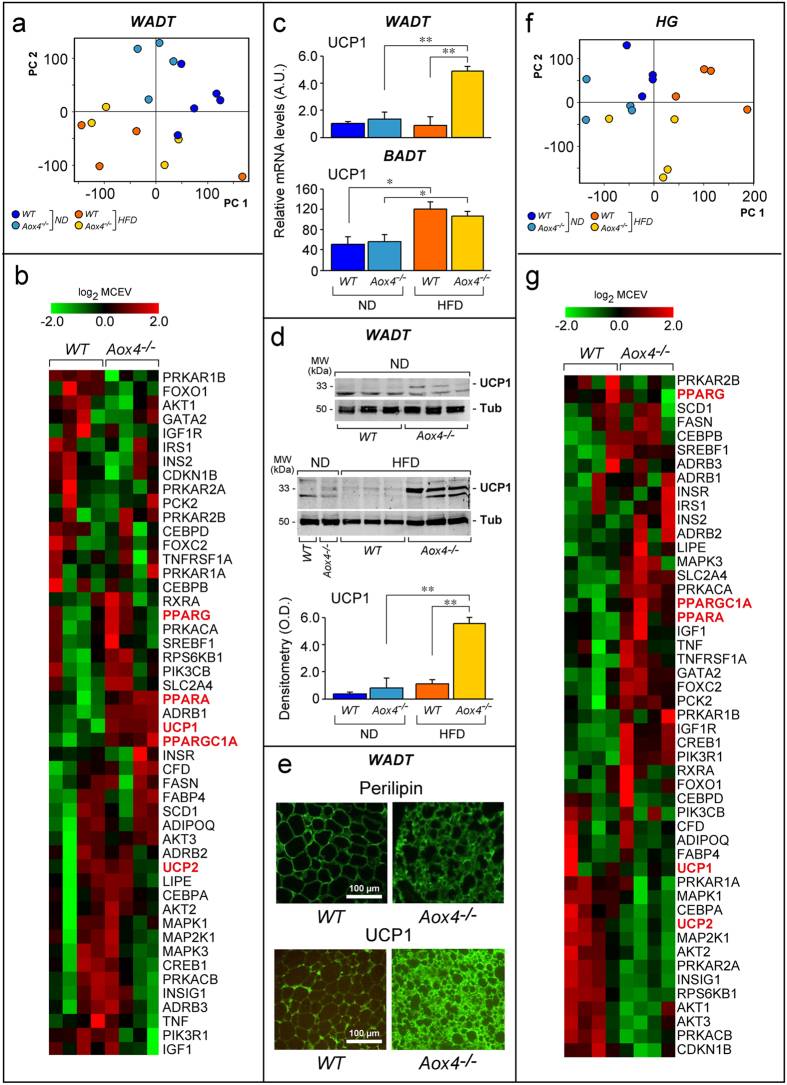
Brown adipocyte gene expression pathways in WADT and HG of *Aox4*^*−/−*^ mice. (**a**,**f**) Principal component analysis (PC) of the gene expression profiles in *WADT* (**a**) and *HG* (**f**) of female *WT* and *Aox4*^*−/−*^ mice subjected to *ND* and *HFD* for 110 days. (**b**,**g**) The heat maps of the indicated genes (METACORE “PPAR regulation of lipid metabolism” pathway) expressed in *WADT* (**b**) and *HG* (**g**) from mice fed *HFD* are shown. Each lane represents a separate animal. Data are shown as the log_2_ of the median centered expression values (MCEV). Genes marked in red are relevant for brown adipocyte homeostasis. (**c**) The graphs illustrate the expression of the *Ucp1* mRNA in the *WADT* (upper graph) and intra-scapular *BADT* of *Aox4*^*−/−*^ and *WT* animals exposed to *ND* and *HFD* for 1 month, as determined by a specific Taqman assay. Values are the mean ± SE of 4 mice. (**d**) The panels show Western-blot analyses of the *WADT* UCP1 protein (ZT = 4) in *Aox4*^*−/−*^ and *WT* animals. For *ND* samples, 40 μg of mitochondrial extracts were used, while 20 μg were used in the case of *HFD* samples. A densitometric quantitation of the Western blot results is illustrated by the lower bar graph following normalization for the tubulin signal. Each lane represents a single animal. The results are expressed as the mean ± S.E. (**e**) The pictures show representative fluorescence micrographs (magnification x200) of *WADT* adipocytes obtained from mice fed *HFD*, after staining for perilipin and UCP1 as indicated. *Significantly different (Student’s t-test, p < 0.05). **Significantly different (Student’s t-test, p < 0.01).

We performed gene-expression microarray studies in visceral *WADT* of female *ND* and *HFD* fed mice. Principal component analysis (PCA) indicates stronger effects of diet than *Aox4-*deletion (Fig. 7a). While 601 probes are differentially expressed (log_2_ fold-change ±0.5, p < 0.005) in *Aox4*^*−/−*^relative to *WT* mice exposed to *ND* (Suppl. Table S1), the number is reduced to 29 (up-regulated = 25; down-regulated = 4) upon *HFD* (Suppl. Table S3). Noticeably, five (*Dbp*, *Hlf*, *Srlc5a6*, *Tef* and *Arntl*) of the 29 genes are clock related genes.

Analysis of the genes selectively modulated in *HFD* fed *Aox4*^*−/−*^mice demonstrates enrichment of 10 pathways (Suppl. Table S3). Four of them (“BMP7 in brown adipocyte differentiation”, “Beta adrenergic receptors in brown adipocyte differentiation”, “Insulin, IGF-1 and TNF-alpha in brown adipocyte differentiation” and “PPAR regulation of lipid metabolism”) are overlapping and contain genes, like *Ucp1*, *Ucp2*, *Ppargc1a* and *Pparα* (Fig. 7b). These genes are typically expressed in brown-adipose-tissue (*BADT*), which controls thermogenesis *via* uncoupling of mitochondrial oxidative phosphorylation^32^. All these genes are also markers of *WADT* trans-differentiation into recruitable-*BADT* (*rBADT*)^33^ and they tend to be up-regulated in *Aox4*^*−/−*^*WADT* (Fig. 7b). Equally up-regulated are various genes of relevance for *BADT* homeostasis^34^ like *Cidea*, *Otop1*, *Cox8b, Elovl3 Cox7a1* and *Ucp1* mRNAs (Suppl. Fig. S10). We focussed our attention on UCP1, as it is a key functional enzyme in *rBADT* uncoupling mitochondrial oxidative phosphorylation and increasing thermogenesis at the expense of fat deposition. We measured the levels of the *Ucp1* mRNA by RT-PCR confirming and extending the microarray results (Fig. 7c). *Ucp1* mRNA levels tend to be higher in *Aox4*^*−/−*^ than *WT* animals upon *ND* feeding. The phenomenon becomes evident in *Aox4*^*−/−*^ mice exposed to *HFD*. In *ND* fed animals, a similar trend is observed also in the case of the UCP1 protein (Fig. 7d). As expected on the basis of the mRNA data, the increase in UCP1 protein levels is evident only in WADT of *Aox4*^*−/−*^ animals fed with *HFD* (Fig. 7d). Induction of UCP1 in *Aox4*^*−/−*^ mice seems to be specific for *WADT*. In fact, the levels of *Ucp1* mRNA determined in intra-scapular *BADT* are similar in *ND*-fed *Aox4*^*−/−*^ and *WT* mice. In addition, the mRNA is equally induced in the two types of animals exposed to *HFD* (Fig. 7c). The presence of *rBADT* cells in *Aox4*^*−/−*^ *WADT* is supported by perilipin (lipid vesicle marker) immune-fluorescence data (Fig. 7e). The morphology of *Aox4*^*−/−*^ adipocytes is consistent with a multi-locular arrangement of lipid vesicles which is typical of *rBADT*. Immunocytochemistry data indicate that UCP1 localizes in multi-vescicular and perilipin-positive adipocytes (Fig. 7e). Taken together, the data support browning of *Aox4*^*−/−*^ *WADT* following exposure to *HFD* and accumulation of UCP1-positive *rBADT* adipocytes which are likely to cause increased local energy-dissipation at the expense of fat accumulation^35^.

The mitochondrial respiratory chain enzymatic activities measured in *WADT* of animals exposed to *ND* and *HFD* are consistent with the browning effect observed in *Aox4*^*−/−*^ mice (Suppl. Fig. S11). *HFD* causes a substantial elevation of citrate synthase activity in *Aox4*^*−/−*^ animals. This supports an increase in the abundance of mitochondria^36^,^37^, which are known to be more numerous and active in *BADT* than *WADT*. A similar increase is not evident in *WT* animals whose citrate synthase levels are similar to *ND* fed *Aox4*^*−/−*^ mice. In addition, the data on complex-I to -V in mitochondria are in line with the UCP1 induction observed in *HFD* fed *Aox4*^*−/−*^ mice. Indeed, *ND* fed *WT* and *Aox4*^*−/−*^ animals are characterized by similar levels of complex-I/-IV specific activities. These enzymatic activities are induced in both *HFD* fed *WT* and *Aox4*^*−/−*^ animals as a consequence of the expected increase in fatty acid oxidation. Significantly, the mitochondrial fractions of *HFD* fed *Aox4*^*−/−*^ mice do not show the induction of complex-V observed in *WT* animals, confirming uncoupling of oxidative phosphorylation consequent to UCP1 induction in *WADT* of *Aox4*^*−/−*^ mice.

### HG gene-expression in HFD fed *Aox4* ^−/−^ mice confirm AOX4 control of lipid metabolism and energy balance

We performed gene-expression studies in *HG* of *HFD*-fed *Aox4*^*−/−*^ and *WT* mice. PCA demonstrates that diet and *Aox4*-deletion cause large effects on gene-expression (Fig. 7f). While 759 probes are differentially expressed (log_2_ fold-change ±0.5; p < 0.005) in *Aox4*^*−/−*^ relative to *WT* mice fed *ND* (Suppl. Table S1), the number is increased to 2,205 (up-regulated = 674; down-regulated = 1531) upon *HFD* (Suppl. Table S3). Relative to *WT* mice, 8 of the 42 genes in the “Circadian-rhythms” pathway are up- (*Camk2d*; *Rora*) or down-regulated (*Arntl*; *Calm1*; *Raf1*; *Calm2*; *Mapk1*; *Map2k1*) in *Aox4*^*−/−*^ *HG*.

In *HG* of *Aox4*^*−/−*^ mice fed *HFD*, 195 pathways controlling different cell processes are enriched (Suppl. Table S3). “Regulation of lipid metabolism_Insulin signaling:generic cascades” stands out, as it supports the role of AOX4 in the control of lipid homeostasis. Enrichment of the “Insulin, IGF-1 and TNF-alpha in brown adipocyte differentiation” is consistent with activation of a *rBADT* phenotype and stimulation of lipid oxidation *via* the mitochondrial pathway in *Aox4*^*−/−*^ mice (Fig. 7g).

### *Aox4* ^−/−^ animals subjected to HFD show resistance to liver steatosis

**Figure 8:**
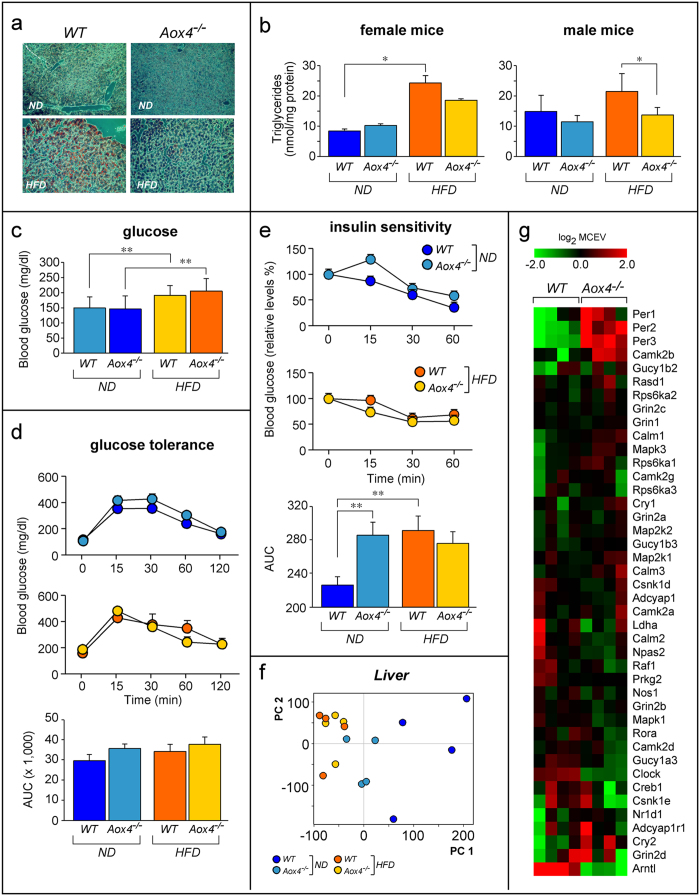
Liver steatosis, insulin sensitivity and liver circadian-rhythm gene expression. Female *Aox4*^*−/−*^ and *WT* animals were fed *ND* or *HFD* for 2 months. (**a**) The panels show representative microscopic fields (magnification x100) of hepatic tissue after staining with oil red and hematoxylin/eosin. (**b**) The bar graphs show the levels of liver triglycerides in female and male animals. Values are the mean ± SE of 5 mice (**c**) The graph illustrates serum glucose levels. Values are the mean ± SE of 10 male mice. (**d**) The line graphs show glucose tolerance curves. The column graphs indicate the values obtained after calculation of the Area Under the Curve (AUC) of the glucose tolerance curves. Values are the mean ± SE of 8 mice. (**e**) The line graphs represent insulin sensitivity curves. The results are expressed in % of the glucose levels determined in *Aox4*^*−/−*^ and *WT* animals at time 0. The column graphs indicate the values obtained after calculation of the Area Under the Curve (AUC) of the insulin sensitivity curves determined for each mouse. Values are the mean ± SE of 10 female mice under *ND* and 6 female mice under *HFD*. (**f**) The panel shows principal component analysis (PC) of liver gene expression profiles in mice fed *ND* and *HFD*. (**g**) The panel shows the heat map for the expression of the METACORE “Circadian Rhythm” genes from female mice fed *HFD*. Each lane represents a separate animal. Data are shown as the log_2_ of the median centered expression values (MCEV). Significantly different; *(Student’s t-test, p < 0.05); **(Student’s t-test, p < 0.01).

The obesity-associated metabolic syndrome includes hepatic steatosis and altered glucose or insulin sensitivity^38^. Histochemical analysis of female liver slices demonstrates that neither *WT* nor *Aox4*^*−/−*^ mice fed *ND* show signs of steatosis. Upon *HFD*, a consistent increase in oil-red-positive hepatocytes is observed in *WT* mice (Fig. 8a). The steatotic response is strongly reduced in *Aox4*^*−/−*^ mice. In line with this, *HFD* causes triglyceride accumulation in female *WT* animals (Fig. 8b). An increase in triglycerides is observed also in *Aox4*^*−/−*^ liver, although it never reaches statistical significance. These last differential effects on triglyceride accumulation are much more evident in male *Aox4*^*−/−*^ mice.

In *ND*-fed *WT* and *Aox4*^*−/−*^ mice, blood glucose basal levels are similar (Fig. 8c). Exposure to *HFD* increases glucose in both types of mice. Nevertheless, the glucose-tolerance curves calculated for *Aox4*^*−/−*^ and *WT* mice are similar regardless of diet and the relative AUC (Area Under the Curve) values are also similar (Fig. 8d). The results of the insulin tolerance tests suggest that *ND*-fed *Aox4*^*−/−*^ animals show a mild and short-lived increase in insulin resistance relative to *WT* mice, which translates into higher AUC values (Fig. 8e). As a consequence, the increase in the AUC values caused by *HFD* (ΔAUC, *HFD*-*ND*) in *WT* mice (Mean of *WT/*ΔAUC = ±66, SE = 4, p-value = 0.007) is no more observed in the *Aox4*^*−/−*^ counterparts (Mean of *Aox4*^*−/−*^*/*ΔAUC = −10, SE = 1, p-value = 0.623). We defined whether the mild insulin resistance observed in *ND*-fed *Aox4*^*−/−*^ mice is accompanied by alterations in the phosphorylation of the insulin receptor (IR) in *WADT* and the downstream intracellular effector AKT in liver and muscles following challenge with insulin^39^. In *WADT*, insulin treatment causes a rapid increase in the phosphorylation levels of IR, while insulin-dependent phosphorylation of AKT is observed in liver and muscles (Suppl. Fig. S12). However, AOX4 deletion does not have a significant effect on the insulin-dependent phosphorylation of the two proteins. The data indicate that the mild insulin resistance observed in *ND*-fed knock-out mice cannot be explained by alterations in the phosphorylation/activation of IR and AKT in three major insulin-responsive tissues.

Gene-expression studies were performed in liver of *HFD* fed *Aox4*^*−/−*^ and *WT* mice. PCA demonstrates that *HFD* exerts selective effects in *Aox4*^*−/−*^ mice (Fig. 8f). While 962 probes are differentially expressed (log_2_ fold-change ±0.5; p < 0.005) in *Aox4*^*−/−*^ relative to *WT* mice exposed to *ND* (Suppl. Table S1), they are decreased to 325 (up-regulated = 248; down-regulated = 77) in *HFD* fed *Aox4*^*−/−*^ mice (Suppl. Table S3). *HFD* up-regulates *Per2* and *Per3* and down-regulates *Arntl* and *Clock* in *Aox4*^*−/−*^ liver. This results in enrichment of the “Circadian-rhythm” pathway (Fig. 8g and Suppl. Table S3). Given the small number of genes selectively modulated by *HFD* in *Aox4*^*−/−*^ liver, only six gene pathways are enriched and five of them control cell-cycle and proliferation. These cell pathways have no obvious links with steatosis. However, it is possible that resistance to steatosis is consequent to the basal or constitutive alterations in gene-expression already observed in the liver of *Aox4*^*−/−*^ animals fed *ND*. In fact, the top (Insulin signaling:generic cascades) and the 6th (Role of Adiponectin in regulation of metabolism) enriched pathways in *Aox4*^*−/−*^ liver are involved in the control of lipid metabolism (Suppl. Table S1).

## Discussion

*Aox4*^*−/−*^ mice are characterized by major alterations in the global gene-expression profile of *HG*, affecting numerous cellular processes. One of the gene-sets which is mostly affected by *Aox4*-deletion controls circadian-rhythms. In *HG*, *Aox4*-deletion modifies the amplitude of the diurnal oscillations of various clock-genes. Given the daily fluctuations of *HG* AOX4 itself, physiological substrate(s)/product(s) of the enzyme may be part of a circuit controlling diurnal rhythms. In *HG,* ATRA may be one such AOX4 product^3^, as the retinoid levels are reduced in the gland of *Aox4*^*−/−*^ mice^14^. In addition, ATRA is a potential regulator of circadian-rhythms^40^ and transcription of *Arntl* and *Per1* clock-genes is controlled by the retinoid *via* nuclear retinoic acid receptors and *Rorα* (retinoic-acid-receptor-Related-Orphan-Receptor-α)^40^,^41^.

The absence of AOX4 in *HG* has systemic consequences, since it influences clock-gene expression also in *WADT* and liver, two tissues devoid of the enzyme. *Aox4*^*−/−*^ mice are characterized by two major systemic changes associated with and potentially determined by clock-genes perturbations, *i.e.* resistance to obesity^42^ and reduction of locomotor activity^43^. Resistance to obesity is due to effects on adipose tissue homeostasis, as *Aox4*^*−/−*^ mice present with lower volumes of *WADT* than *WT* animals. If *Aox4*^*−/−*^ mice are fed *HFD*, the effect is magnified and accompanied by protection from liver steatosis. Resistance to obesity and steatosis is due to decreased feed efficiency and it is not directly related to alterations in locomotor activity, appetite or food intake. It is well known that circadian rhythms, locomotor activity and obesity are tightly related physiological processes^27^,^28^. Indeed, *Clock* knock-out mice are characterized by an altered locomotor activity pattern which is similar to the one observed in our *Aox4*^*−/−*^ mice^44^. However, *Clock* knock-out mice are hyperphagic and obese, which is the opposite of what we observe in our animals. Thus, alterations in *Clock* expression may be at the basis of the decrease in locomotor activity of *Aox4*^*−/−*^ animals, while the gene is unlikely to play a role in resistance to obesity. *Rorα* is another clock gene that may play a role in fat deposition, since it has been linked to reduced adiposity, resistance to diet-induced obesity, protection against hepatic steatosis and improved glucose homeostasis^40^,^45^,^46^,^47^. The intensity of *Rorα* diurnal oscillations is reduced in *HG* of both female and male animals, while a similar trend is observed only in female *WADT* and liver (Figs 1 and 2). These data suggest a possible involvement in the systemic effects of AOX4 on fat deposition. However, our results do not support this hypothesis, as we found no enrichment (Suppl. Table S4) of the genes regulated in *Aox4*^*−/−*^ mice among *Rorα* direct targets^48^.

Sex has been shown to influence circadian-rhythms, fat deposition^49^, locomotor activity as well as *Aox4*, *Aox3* and *Aox1* expression^15^. *Aox4*^*−/−*^ animals show resistance to diet induced obesity regardless of sex. By the same token, the overall decrease in locomotor activity shows no sex-specificity. Reduction in the oscillatory amplitude of clock-genes is the only trait of *Aox4*^*−/−*^ animals which is influenced by sex, albeit in a tissue-specific fashion. In fact, this parameter is significantly altered in the *HG* and hypothalamus of both female and male *Aox4*^*−/−*^ mice. In contrast, only female *Aox4*^*−/−*^ *WADT* and liver show a diminished diurnal oscillatory amplitude of *Per2*, *Dbp* and *Arntl* expression. A similar phenomenon is observed in the case of hepatic *Rorα*. Given up-regulation of AOX3 and AOX1 proteins in liver and *WADT* of male *Aox4*^*−/−*^ mice, we propose that the oscillatory behaviour of clock-genes in different tissues is controlled by common substrate(s)/product(s) of AOX4, AOX3 and AOX1 isoenzymes.

In *WADT*, *HG* AOX4 controls fat deposition by modulation of the balance between lipid synthesis and oxidation. One of the factors controlling this balance is represented by the relative proportion of cells presenting with the characteristics of *WADT* and r*BADT* adipocytes^50^, which are responsible for non-shivering thermogenesis. r*BADT* adipocytes contain many mitochondria and high levels of UCP1, which uncouples oxidative phosphorylation. In *Aox4*^*−/−*^ *WADT*, *HFD* up-regulates *Ucp1* and other r*BADT* markers as well as genes controlling mitochondrial activity. Thus, AOX4 absence in *HG* determines a functional switch of white into brown adipocytes which increases burning and reduces deposition of fat. The increased number of r*BADT* adypocytes in *Aox4*^*−/−*^ *WADT* does not affect whole-body energy-consumption, as assessed by indirect calorimetric analysis, although changes may go unnoticed for at least two reasons. First, we calculated that an approximately 3% energy imbalance would be sufficient to produce the differences in weight gain between *Aox4*^*−/−*^ and *WT* mice^51^. Second, *Aox4*^*−/−*^ mice show increased energy expenditure relative to *WT* controls, if decreased locomotor activity is taken into account. Lack of systemic alterations in energy balance are accompanied by a lack of effects on body temperature, which is not different in *Aox4*^*−/−*^ and *WT* animals at any of the zeitgebers considered (Suppl. Fig. S13).

In a previous study we reported that *Aox4*^*−/−*^ mice are characterized by alterations in the thickness and structure of the skin^14^. In addition, *HG* secretions are purported to play a role in thermal insulation of the skin^17^. This may suggest that *WADT* browning of *Aox4*^*−/−*^ animals may be stimulated to compensate heat loss due to improper skin insulation. However our data are against this hypothesis, as indicated by the results obtained in female animals maintained under thermoneutral conditions (30 °C) for 30 days (Suppl. Fig. S14). In these conditions, we determined the abdominal fat volume (MRI analysis) in animals kept under ND. As an internal control of our experiment, we compared *Ucp1* mRNA in *BADT* of animals maintained at 22 °C and at 30 °C. As expected, BADT *Ucp1* mRNA levels are lower in *WT* animals maintained at 30 °C relative to what is observed at 22 °C (Suppl. Fig. S14a). This is in line with the decrease in thermogenic activity expected for mice under thermoneutral conditions. Significantly, a similar decrease is observed also in *Aox4*^*−/−*^ mice, which is against the hypothesis that improper skin insulation may be at the basis of the observed effects on fat deposition. More importantly, the lower volume of abdominal fat in *Aox4*^*−/−*^ mice relative to the *WT* counterparts that was already observed at 22 °C (see Fig. 6b, time 1 and 2) is unaffected by the switch to 30 °C (Suppl. Fig. S14b). During the observation period no significant difference in body weight between WT and *Aox4*^*−/−*^ mice maintained at 30 °C was observed (Suppl. Fig. 14c). Taken together, these data further support the idea that browning of WAT and UCP1-dependent thermogenesis are not stimulated to compensate heat loss due to improper skin insulation in *Aox4*^*−/−*^ mice.

As for the molecular mechanisms underlying *WADT* browning in *Aox4*^*−/−*^ mice, circulating factors produced in *HG*, such as Irisin, which drives *BADT* development^52^ may contribute to the process. In fact, *Fndc5* (coding for the Irisin precursor) expression is higher in *Aox4*^*−/−*^ than *WT HGs*, regardless of diet (Suppl. Tables S1–3). Alterations in the circulating or local levels of substrates or products of AOX4 enzymatic activity in *HG* may also participate in the process of *WADT* browning. Retinaldehyde (RAL), a known AOX substrate, and ATRA, its oxidation product, may be candidates^10^. In fact, ATRA and RAL control trans-differentiation of *WADT* into *rBADT*^53^. However, indirect data are against an involvement of circulating ATRA deriving from *HG* AOX4 activity in the process (Suppl. Fig. S15). In fact, no significant difference in serum ATRA is observed between female *Aox4*^*−/−*^ and *WT* mice at any of the *ZT* considered. In addition, male knock-out mice present with significantly higher levels of blood ATRA than the *WT* counterparts at *ZT*4. Collectively, these data indicate that other enzymes, i.e retinaldehyde dehydrogenases^54^,^55^, play a more important role in the control of serum ATRA levels than *HG* AOX4, liver AOX3 or *WADT* AOX1. As for the local production of ATRA by AOX3 and AOX1, it is equally unlikely that they are involved in the fat accumulation deficits observed in liver and *WADT* of *Aox4*^*−/−*^ animals. In fact, sex controls the levels of liver AOX3 and *WADT* AOX1 in knock-out mice, whereas it does not affect resistance to obesity. In *Aox4*^*−/−*^ liver, the mechanisms underlying resistance to steatosis are unknown, although the gene-expression and metabolomic data are in line with decreased *HFD*-induced lipid synthesis/accumulation. Protection from *HFD*-induced steatosis may involve immune responses, as indicated by pathway enrichment analysis (Suppl. Table S3). This may reflect modulation of the chronic metabolic-related inflammation (meta-inflammation) associated with obesity^56^ which may influence the secretion of soluble factors controlling liver steatosis^57^.

Resistance to diet-induced obesity and liver statosis are often accompanied by increased glucose tolerance and insulin sensitivity^58^. Our insulin tolerance tests suggest that *Aox4*^*−/−*^ animals show signs of mild insulin resistance. However, the insulin-dependent phosphorylation of IR or AKT at the level of the three major insulin-responsive organs, liver, *WADT* and muscles do not explain these results. Hence, we looked for other underlying mechanisms, performing a detailed METACORE analysis on our microarray data using the broadest version of this pathway (“Development Regulation_Insulin pathway). We compared the genes of this pathway differentially expressed in the *HD*, liver and *WADT* of *Aox4*^*−/−*^ and *WT* animals fed *ND* and *HFD* (Suppl. Fig. S16). This analysis demonstrates the already mentioned up-regulation of UCP1 in *WADT* of *Aox4*^*−/−*^ animals fed *HFD*. Finally, it indicates that the hormone-sensitive-lipase, LIPS or LIPE, which is involved in the activation of *BADT* genes, is up-regulated in liver of *HFD* fed *Aox4*^*−/−*^ mice. Overall the data support the idea that *Aox4* deletion exerts significant influences only on the expression of components of the insulin pathway playing a role in *BADT* homeostasis and are predominantly observed in the *WADT* of *HFD* animals.

Given the relevance of the tryptophan/serotonin pathway for circadian-rhythms^59^,^60^ and fat deposition, the finding that tryptophan and 5HIAA are two novel physiological substrates of *HG* AOX4 and liver AOX3, is of biological significance. In fact, it is possible that variations in *HG*/blood levels of serotonin, tryptophan, 5HIAA or AOX-derived metabolites contribute to the systemic effects of *Aox4*-deletion on clock-genes. Interestingly, a very recent study demonstrates that knock-out mice for peripheral tryptophan-hydroxylase-1 (TPH1) are characterized by resistance to obesity which is explained by an increase in brown adipose tissue thermogenesis^61^. In addition, mice deficient in neuronal TPH2 show altered thermogenesis and behavior^59^. With respect to this, it should be noticed that the levels of *HG* serotonin are significantly lower in *Aox4*^*−/−*^ than *WT* mice (*Aox4*^*−/−*^: mean ± SE = 459 ± 38 fmol/mg tissue, N = 6; *WT*: mean ± SE = 574 ± 39 fmol/mg tissue, N = 6, t-test = p < 0.05). In serum, a similar trend is observed (*Aox4*^*−/−*^: mean ± SE = 4.3 ± 0.9 nmol/ml, N = 6; *WT*: mean ± SE = 6.1 ± 0.3 nmol/ml, N = 6), although the results do not reach statistical significance. These data suggest a possible involvement of *HG* serotonin in the modifications of circadian rhythms and resistance to obesity observed in our experimental model.

In conclusion, the study provides insights into the physiological function of AOX4 demonstrating that the enzyme plays a role in the control of diurnal rhythms, adipogenesis and locomotor activity, supporting the link between these three processes. Since the *Aox4*^*−/−*^ model was generated on a background strain that lacks melatonin, our data imply that one or more AOX4 products or substrates other than melatonin mediate alterations in the biological clocks. The reported effects on diurnal rhythms, fat deposition and locomotor activity do not impact life expectancy, as *Aox4*^*−/−*^ and *WT* mice show the same survival curves (Suppl. Fig. S17). The involvement of AOX4 in the process of fat deposition may also be of relevance for the human situation, as human AOX1 may play a similar role as mouse AOX4 in adipogenesis^62^.

## Methods

### Animals and housing

All the procedures involving animals and their care were conducted in conformity with the institutional guidelines in compliance with the national (Legislative Decree n. 26, March 4, 2014; Authorization n.19/2008-A issued March 6, 2008, by the Italian Ministry of Health) and international law and policies; EU directives and guidelines (EEC Council Directive 2010/63/EU); the NIH Guide for the care and use of laboratory animals (2011 edition). All the animal experiments performed in this study were validated by the Institutional Ethical Review Committee for the Animal Care and Use (ACU) and approved by the Italian “Istituto Superiore di Sanità” under the Project “Regolazione *in vivo* dell’espressione di geni. Importanza e ruolo delle aldeidi ossidasi nella regolazione dell’adipogenesi, del metabolismo, dei ritmi circadiani e dell’attività motoria in topi knock-out per geni codificanti la famiglia delle molibdo-flavoproteine” (RS S04_02, April 2010).

*Aox4*^*−/−*^ mice were created on a C57BL/6N × sv129 genetic background^14^ and were backcrossed for 10 generations with the C57BL/6N lineage. The mice used in the present study were homozygous male and female *AOX4*^*−/−*^ and their wild-type (*WT*) littermates. For experimental design settings, to avoid genetic drift, male and female mice were selected by interbreeding heterozygous (*Aox4*^+/−^) to generate *Aox4*^*−/−*^ and *WT* littermates. Animals were maintained in a pathogen-free animal facility at 22 °C under a 12-h light/12-h dark cycle with light on at 7:00 a.m. Animals had free access to water and standard chow (*ND*, 2918; Harlan Teklad Global Diet, Madison, WI) or a high-fat diet (*HFD*, 42% kcal in fat, TD88137). All the experiments were carried out in 10–22 weeks-old female and male mice, unless otherwise specified. All treatment groups were weight matched and randomized to treatment at the initiation of the experiments.

### Wire hanging test

Fourteen-week-old *Aox4*^*−/−*^ (n = 9) and *WT* (n = 8) female mice were used for the experiment. A similar experiment was conducted on male animals. In this case, 9 *Aox4*^*−/−*^ and 9 *WT* mice were used and the data recorded at seven and fourteen weeks of age. Animals were suspended by their forelimbs to a 1.5 mm thick, 60 cm long metallic wire suspended 45 cm above soft ground, and the chronograph was started. The chronograph was stopped anytime the animal fell and restarted when it was placed again on the wire; the test was stopped after 180 seconds of suspension. The number of falls from the wire was recorded; after 10 falls, the test was discontinued. At the beginning of the test, each animal was given a score of 10 that was reduced by 1 after each fall. Results were expressed as the ‘average score’. The average score was calculated at any time point of the test as (10n–x)/n where n was the number of animals of the tested strain and x the cumulated number of falls.

### Determination of muscle fibres diameter

Tibial muscles of age matched female and male *Aox4*^*−/−*^ or *WT* animals (3 individual mice/experimental group) were isolated and frozen. Following staining with eosin, minimal Feret’s diameter was measured according to the protocol available in Treat-NMD Neuromuscular Network [SOP (ID) number: MDC1A_M.1.2.002] WEBsite (http://www.treat-nmd.eu/research/overview). At least 500 fibers were examined for each specimen.

### Indirect calorimetry

Oxygen consumption and carbon dioxide production were measured by indirect calorimetry using an open-circuit system. The system is made of 2 cage/respiratory chambers and of a blank cage used as a reference line. The differential measurements of O_2_ and CO_2_ compared with the reference line were performed every 7 min for each respiratory chamber with the use of 2 paramagnetic O_2_ analyzer and of 2 infrared CO_2_ analyzer. The air flow to the chambers was 1.5 liters/min. Energy expenditure was calculated from the daily total volumes of oxygen uptake and of carbon dioxide produced^63^,^64^, using the following equation: (16.07*VO_2_) + (4.69*VCO_2_). Respiratory Quotient (RQ) was calculated as CO_2_ production/O_2_ consumption.

Two different indirect calorimetry experiments were performed on twenty-week-old *Aox4*^−/−^ (n = 20) and *WT* (n = 20) female animals, housed in groups of 4 mice per cage/respiratory chamber (5 cages per experimental group). In the first experiment, respiratory exchanges were measured on animals fed *ND*, while the experiment was performed after two weeks of adaptation to *HFD*. During the whole experimental period, mice were housed at a temperature of 22.0 ± 1.0 °C under a 12 h light-dark cycle (07:00–19:00 h). Food and water were available *ad libitum*. Animals were adapted to the respiratory chambers by placing them in the cage systems for 48 h before starting data collection. Body weights of animals were determined at the beginning and at the end of the respiratory exchanges trials. In both experiments, each group of animals was subjected twice to respiratory exchanges trial (2 consecutive cycles of 24 h), with 1 month in between, carried out using the one or the other respiratory chamber (4 cycles of 24 h for each cage/respiratory chamber). Oxygen consumption, carbon dioxide production, RQ end energy expenditure were expressed for the light phase (day, 07:00–19:00 h), dark phase (night, 19:00–07:00 h) and the entire 24 h period. At the end of the experiment blood samples, *HG*, liver, visceral, subcutaneous and inguinal adipose tissues were removed and analysed.

### Locomotor activity

Seven- and ten-week-old male as well as female mice fed *ND* were placed individually in standard mouse cages equipped with infrared sensors to detect locomotor activity. In the case of males, 6 *Aox4*^*−/−*^ and 6 *WT* mice were maintained on a 12:12 LD cycle for one week, then the light was switched off and constant darkness (12:12 DD) maintained for 6 weeks. The locomotor activity of male animals was monitored every 6 minutes. In the case of female mice, the experiment locomotor activity was monitored every 15 min for 2 weeks. Body weight, temperature and food intake were measured twice a week.

### High fat diet

Eight-ten-weeks old male and female mice were used. 20 *Aox4*^*−/−*^ and 20 *WT* were randomized and divided in two groups. 10 *Aox4*^*−/−*^ and 10 *WT* mice were fed standard chow (*ND*) while 10 *Aox4*^*−/−*^ and 10 *WT* animals were fed with a high fat diet (*HFD,* Teklad TD.88137). Food intake and body weight were measured twice a week. At the end of experiments, animals were sacrificed and blood, *HG*, adipose tissues as well as liver were isolated. Serum glucose, NEFA (non-esterified fatty acids), triglycerides and cholesterol were measured according to standard methods routinely used for human clinical samples. Total triglyceride content in tissues was measured using Adipogenesis Kit (Sigma-Aldrich).

### Glucose and Insulin Tolerance Tests

Female mice approximately (16 weeks of age) were treated as indicated^65^. For glucose tolerance tests, mice were injected intra-peritoneally with glucose (2.0 g/kg body weight) or insulin (750 milliunits/Kg body weight). Tail blood glucose was measured at various time points using a glucometer (Bayer).

### Serotonin measurement

The levels of serotonin in *HG* and serum were determined on 6 separate *Aox4*^*−/−*^ and *WT* animals with an HPLC-based assay according to Invernizzi *et al.*^66^.

### *In vivo* MRI

Twelve-week-old female *WT* mice (n = 6) and the same number of age-matched *Aox4*^*−/−*^ animals were randomly recruited to determine the fat accumulation by MRI analysis. MRI experiment were carried out one day before the onset of the experiment (T0), one month (T1) and two months (T2) after the starting *HFD*. Before MRI experiments, mice were anaesthetized by inhalation of a mixture of O_2_ (30%) and NO_2_ (70%) containing 3% isoflurane. Mice were laid prone to a small glass column “Animal Bed- Brucker BioSpec Systems” Brucker connected to an anaesthetic system. During the whole duration of experiments the percentage of isoflurane was maintained from 0.8 to 1.2. Respiratory frequency was monitored by a mechano-sensor placed on the chest. All analyses were carried in a range of respiratory activity from 50 to 90 breaths/min.

All MRI experiments were carried out using a Brucker Biospec 70/30, equipped with a Brucker BGA12 gradients insert. A 72-mm transmitter/receiver birdcage coil was used. After a coronal scout SE image, 20 coronal multislice T1-weighted (T1W) images were acquired to localize the area of interest. All images were acquired with the following parameters: TR = 782.5 ms, TE = 14.3 ms, FOV = 30 × 30 cm 2, matrix size = 512 × 256, slice thickness = 2 mm, NEX = 1. All experiment were done by removing the sat-faturation process. To reduce image artefacts, a respiration trigger system was adopted. The body weight was recorded just before MRI analysis.

All images were exported as DICOM (Digital Imaging and COmmunications in Medicine) files. This version is required to maintain the original size of MRI acquisition. A public Java-based image processing program (Image J) was utilized for morphometric analysis. Total fat volume measurements were carried out by selecting five serial slices starting from the first section (1 cm of thickness) below the diaphragm to the hypogastric region. For each single section the total area was first determined. Fat accumulation (light signal) was investigated by measuring extraperitoneal (subcutaneous) and intraperitoneal (visceral). The volume was determined by multiplying the area of interest (mm^2^) by the thickness of sections (2 mm). The ratio between the fat volume and the whole volume of each single corresponding section was also calculated.

### RT-PCR, Immunocytochemistry and Western blot analysis

Total RNA was isolated from indicated tissues or cells with TRIzol reagent (Invitrogen), and the cDNA was synthesized using Reverse Transcription System (Promega). For the histological and immune-cytochemistry studies, tissues were fixed with Z-Fix (Anatech Ltd.), embedded in paraffin, sectioned at a thickness of 6–8 μm, and stained with hematoxylin and eosin. Immunostaining experiments in the adipose tissue were conducted with an anti-perilipin antibody (D418, Cell Signaling) and anti-UCP1 (ab10983, Abcam). Western blots were performed using specific anti-UCP1 (ab23841, Abcam), anti-α-tubulin (T-5168, Sigma), anti-AOX1, -AOX2, -AOX3 and -AOX4 antibodies^15^. Real-time PCR was performed with Taqman assays according to standard protocols. The list of Taqman assays is present in Supplementary Methods. In all cases normalization of the results was performed with *Mrsp33*, whose levels are not influenced by circadian rhythms and Aox4 deletion in any of the tissues considered.

### Gene-expression and metabolomics

Total RNA from mouse tissues was extracted in the morning (ZT = 1–4) with the miRNeasy Mini kit (QIAGEN), labelled with the Lowinput Quick Amp labelling Kit (Cy3 mono color, Agilent) and hybridized to gene-expression microarrays (Agilent). Fluorescent signals were quantified with an Agilent microarray laser scanner. The microarray data and protocols were deposited in the Arrayexpress database (accession No. E-MTAB-3820). We performed gene enrichment analysis on METACORE-annotated process networks (http://thomsonreuters.com/metacore), using the hypergeometric test after correction for multiple testing and calculation of the false discovery rate (FDR). Differential metabolomics were performed as detailed in Supplementary Methods.

## Additional Information

**How to cite this article**: Terao, M. *et al.* Mouse aldehyde-oxidase-4 controls diurnal rhythms, fat deposition and locomotor activity. *Sci. Rep.* **6**, 30343; doi: 10.1038/srep30343 (2016).

## Supplementary Material

Supplementary Information

Supplementary Table S1

Supplementary Table S2

Supplementary Table S3
