# Supplementary material for: Mouse aldehyde-oxidase-4 controls diurnal rhythms, fat deposition and locomotor activity
Source: Sci Rep. 2016 Jul 26;6:30343. doi: 10.1038/srep30343 (PMC4960552; doi:10.1038/srep30343)
Supplement: Supplementary Information [file srep30343-s1.pdf]

SUPPLEMENTARY INFORMATION

TITLE: Mouse aldehyde-oxidase-4 controls diurnal rhythms, fat deposition and locomotor activity

Mineko Terao[1], Maria Monica Barzago[1], Mami Kurosaki[1], Maddalena Fratelli[1], Marco Bolis[1], Andrea Borsotti[1], Paolo Bigini[2], Edoardo Micotti[3], Mirjana Carli[4], Roberto William Invernizzi[4], Renzo Bagnati[5], Alice Passoni[5] Roberta Pastorelli[6], Laura Brunelli[6], Ivan Toschi[7], Valentina Cesari[7], Seigo Sanoh[8] and Enrico Garattini[1,9].

[1]Laboratory of Molecular Biology and [2]Laboratory of Biochemistry and Protein Chemistry, Department of Molecular Biochemistry and Pharmacology; [3]Laboratory of Neurodegenerative diseases; [4]Laboratory of Neurochemistry and Behaviour, Department of Neuroscience. [5]Analytical Instrumentation Unit and [6]Laboratory of Mass Spectrometry, Department of Environmental Health Sciences; IRCCS-Istituto di Ricerche Farmacologiche "Mario Negri", via La Masa 19, 20156, Milano, Italy. [7]Department of Agricultural and Environmental Sciences; Università degli Studi di Milano, via Celoria 2, 20133 Milano, Italy.

[8]Graduate School of Biochemical and Health Sciences, Hiroshima University, Hiroshima Japan.

[9]Correspondence to: Enrico Garattini, MD, Laboratory of Molecular Biology. IRCCS-Istituto di Ricerche Farmacologiche "Mario Negri", via La Masa 19, 20156, Milano, Italy. Tel. No. +390239014533; e-mail address: enrico.garattini@marionegri.it.

SUPPLEMENTRY METHODS

Circadian cycles

Seventeen *Aox4*$^{-/-}$ and 20 *WT* females (10-12 weeks) were randomized in two groups. In the first group 8 *Aox4*$^{-/-}$ and 10 *WT* mice were maintained in rooms on a 12h light-dark cycle with the light on at 7:00 a.m (12L/12D). In the second group, animals (9 *Aox4*$^{-/-}$ and 10 *WT* mice) were maintained in rooms where the light-dark cycle is inverted (12D/12L). Animals were kept for at least one month to let them get adjusted to the light-dark cycle before measurements of any parameter. The same type of experiment was conducted on 19 male *Aox4*$^{-/-}$ and 25 *WT* mice (14-16 weeks) after randomization in two groups. Body weight was recorded twice a week. After 10 weeks, animals were euthanized. Blood samples, *HD*, liver, hypothalamus, visceral, sub-cutaneous and inguinal adipose tissue were removed and analysed. Three independent experiments were performed on distinct cohorts of animals.

Taqman assays

For the PCR determination of the mRNA species described in the study, the following Taqman assays (Applied Biosystems) were used: AOX1=Mm01255332_m1; AOX3= Mm00508167_m1; *Per2*= Mm00478113_m1; *Dbp*=Mm00497539_m1; *Arntl*= Mm00500226_m1; *Ucp1*= Mm01244861_m1; *Elovl3*=Mm00468164_m1; *Cox7a1*= Mm00438296_m1; *Clock*=Mm00455950; *Rorα*=Mm01173766; *Rev-Erbβ* (*NR1d2*)=Mm00441730; *Mrps33*= Mm03009791_m1. Reverse transcription of total RNA was performed as described (Centritto et al., 2015; Fisher et al., 2015).

Measurement of Melatonin

The HPLC-MS/MS system used for the analysis of melatonin consisted of a 1200 series pumps and autosampler, (Agilent Technologies, Santa Clara, CA) interfaced to an API 5500 triple quadrupole

mass spectrometer, equipped with a turbo ion spray source (AB Sciex, Thornhill, Ontario, Canada). The HPLC separation was obtained with an Atlantis C18 column, 150×2.1 mm, 3.5µm particle size (Waters Corporation, Milford, MA), using an elution mixture composed of solvent A (0.1 % formic acid in water) and solvent B (acetonitrile). The injection volume was 8 µl and the flow rate was 160 µL/min. The elution gradient was from 2 to 62% of solvent B in 12 minutes, then to 99% of solvent B for 2 minutes and re-equilibration for 9 min at 2% of solvent B. The mobile phase was directly introduced into the ion source, which was operated with a turbo ion gas temperature of 400 C°. Kynurenin-D4 was used as the internal standard (IS), at the concentration of 1 ng/mL. An external calibration curve was prepared with variable amounts of melatonin (0, 1, 3, 10 and 30 pg) and a fixed amount of kynurenin-D4 (1,000 pg), in 200 µL of initial HPLC mobile phase. Mass spectrometric analyses were performed using positive ionization and selected reaction monitoring (SRM) mode, measuring the fragmentation products of the protonated pseudo-molecular ions, as shown in the table below which lists SRM transition and collision energies for melatonin and the internal standard kinurenin-D4.

Substance	SRM transitions	Collision Energy
**Melatonin**	233.2 -> 174.1	19
	233.2 -> 130.1	57
**Kinurenin-D4**	213.2 -> 150.1	27
	213.2 -> 80.0	20

Samples (300 µL) were spiked with internal standard (300 pg) and extracted with 3 volumes of acetonitrile (900 µL). After vortexing for 1 min, the samples were centrifuged at 13200 rpm in an Eppendorf centrifuge and the supernatant was transferred to another Eppendorf tube. The tubes were placed in an evaporative centrifuge at 30°C until complete dryness. The residue was reconstituted with 70 µL of initial HPLC mobile phase, centrifuged and transferred to autosampler vial inserts.

Untargeted Metabolomics

<u>Sample Preparation:</u> Harderian glands (HG) from *Aox4*-KO and -WT mice (3 mice/group) were homogenized by sonication using a Branson sonicator (model 250, Branson, Danbury, Conn., USA) in 1mL ice-cold MeOH. Homogenates were centrifuged at 16000xg for 15 minute at 4°C. Supernatants were divided into two aliquots, dried, and then reconstituted in acidic or basic LC-compatible solvent.

<u>Liquid Chromatograophy_tandem mass spectrometry (LC-MS/MS) analysis:</u> A portion (2 microL) of metabolite extract was analysed by LC-MS/MS, using an LTQ Orbitrap XL™ (Thermo Scientific), interfaced with a 1200 series capillary pump (Agilent). The MS instrument was operated in positive (POS) and negative (NEG) ionization modes. Untargeted metabolomic data were processed using the MS label free differential analysis software SIEVE v 2.2 (ThermoFisher). SIEVE was run on all the LC–MS full-scan chromatograms using the component extraction setting. The chromatograms were time-aligned, blank subtracting (solvent background) and referencing the sample acquired in the middle of the sequence. The framing parameters were set at 0.01 Da for the m/z window and 0.35 min for the retention time (RT) window; 500,000 was used as the intensity threshold. Before any statistical analysis the value of each molecular species (frame) detected by SIEVE was normalized to the intensity of the internal standards using the FRAME option for spiked internal standards (all frames are normalized to the designated frame with the internal standard ion). An additional filtering criterion was then applied to include in the dataset only frames with an intensity coefficient of variation <50% in at least one experimental group

<u>Multivariate data analysis:</u> The normalized ion intensity data for each sample was submitted to the SIMCA-P13 software package (Umetrics) for multivariate data analysis. The variables were scaled using Pareto scaling to increase the low abundance ions without significantly amplifying the noise. To maximize class discrimination, the data were analysed by orthogonal partial least-squares discriminant analysis (OPLS-DA). S-plots were calculated to visualize the relationship between

covariance and correlation within the OPLS-DA results. The features that significantly contributed to discrimination between groups were identified.

Identification of metabolites: For metabolite identification, the frame m/z values were used for batch searches on the METLIN database (http://metlin.scrpss.edu) and Human Metabolome Database (HMDB, http://www.hmdb.ca/). Accurate mass data and isotopic distribution for the precursor and product ion were compared to spectral data of the reference compounds in the databases. Lipids were tentatively identified by high mass accuracy and MS/MS fragment ions using the LIPID Mass database without authentic standards. Identifications were reported only for metabolites with accurate mass match < 5 ppm.

Isolation of mitochondria and determination of respiratory chain enzymatic activities

Mitochondrial fractions were prepared from *HD* and *WADT* tissues derived from 6-8 animals per experimental group according to the method described by Frezza *et al.*, 2007. The mitochondrial respiratory chain enzymatic activities of Complexes I-IV and citrate synthase were assessed according to the methods described by Spinazzi *et al.*, 2012, while Complex-V (ATP synthase), was measured according to the protocol described by Barrientos *et al.*, 2009. The crude mitochondrial extracts (8-10 µg of protein) were used in 0.2 ml reaction, and the absorbance was measured in Tecan infinite M200 spectrophotometer (Tecan, Maennedorf, Switzerland). The results obtained are presented as Specific Activities (mmol/min/mg protein).

Protein purification, enzymatic assays and mass-spectrometry

Catalytically active AOX4 and AOX3 were purified from *HG* and liver respectively (Terao et al., 2009). Enzymatic assays on the purified proteins were performed with tryptophan and 5HIAA using the Amplex Red XOR assay kit (Molecular Probes), which measures the formation of H_2O_2. Melatonin and ATRA were also measured by mass-spectrometry.

Mass Spectrometry

The HPLC-MS/MS system used for the analysis of the AOX4 and AOX3 products generated from 5HIAA and tryptophan consisted of a 1200 series pumps and autosampler, (Agilent Technologies, Santa Clara, CA) interfaced to a Q Exactive Orbitrap mass spectrometer, equipped with a HESI source (Thermo Fisher Scientific, Waltham MA). The HPLC separation was obtained with an Atlantis C18 column, 150×2.1 mm, 3.5μm particle size (Waters Corporation, Milford, MA), using an elution mixture composed of solvent A (0.1 % formic acid in water) and solvent B (acetonitrile). The injection volume was 5 μl and the flow rate was 200 μL/min. The elution gradient was from 1 to 50% of solvent B in 16 minutes, then to 99% of solvent B in 2 minutes and re-equilibration for 6 min at 1% of solvent B. The Q Exactive instrument conditions were as follows: polarity: positive, spray voltage: 4000 V, capillary temp.: 320 °C, probe heater temp.: 300 °C, full MS resolution: 70000, scan range: 125 to 600 m/z, MS/MS resolution: 17500, isolation window: 2.0 m/z; normalized collision energy: 35.

Samples (200 – 500 μL) were evaporated in a rotary vacuum centrifuge and reconstituted in 40 μL of water. The mass spectrometric analyses were done in full MS and MS/MS scans of the appropriate pseudo-molecular ions: 205.09715, 221.09207, 192.06552, 208.06043 m/z for tryptophan, hydroxy-tryptophans, 5HIAA and dihydroxyindoleacetic acid, respectively. The substances were identified by extracting their high resolution ion chromatograms at 5 ppm width and their MS/MS spectra at the corresponding retention times. ATRA was measured by mass-spectrometry as already described (Terao et al., 2009).

Insulin-dependent phosphorylation of IR and AKT in the liver, WADT and muscles of WT and $Aox4^{-/-}$ mice

ND-fed *WT* and *$Aox4^{-/-}$* mice were starved overnight and subsequently treated with insulin (750 milliunits/kg) for 15 minutes. Liver, inguinal WADT and muscles (soleus and gastrocnemius) were isolated (Agouni et al., 2010). Total tissue extracts were used to perform Western blot analysis with

the following antibodies: anti-IR (ab69508, Abcam), anti- phosphorylated-IR (ab5678, Abcam), anti-AKT (Cat. No. 9272, Cell Signaling), anti-phosphorylated AKT (Cat. No. 9271, Ser473, Cell Signaling) and anti-tubulin (T5168, Sigma).

Measurement of lipid content in the faeces

The amount of total lipids in the faeces of *WT* and *Aox4*$^{-/-}$ mice was determined according to a standard procedures (Kraus et al., 2015) after extraction with chloroform/methanol (2:1 v/v, Folch Method). Single lipid species were quantitated after thin layer chromatography of the chloroform/methanol extracts on Silica gel 60 TLC plates (Art. 5721, Merck) in hexane/ethyl-ether/acetic acid (80/20/1 v/v) and staining with 50% sulphuric acid at 120°C for 10 minutes (Fuchs et al., 2011).

SUPPLEMENTARY REFERENCES

Agouni, A., Owen, C., Czopek, A., Mody, N. & Delibegovic, M. In vivo differential effects of fasting, re-feeding, insulin and insulin stimulation time course on insulin signaling pathway components in peripheral tissues. Biochem Biophys Res Commun 401, 104-111, doi:S0006-291X(10)01693-1 (2010).

Barrientos, A., Fontanesi, F. and Diaz, F. (2009) Evaluation of the mitochondrial respiratory chain and oxidative phosphorylation system using polarography and spectrophotometric enzyme assays. In Current Protocols of Human Genetics. Chapter Unit 9.3 doi: 10.1002/0471142905.hg1903s63.

Centritto, F., Paroni, G., Bolis, M., Garattini, S.K., Kurosaki, M., Barzago, M.M., Zanetti, A., Fisher, J.N., Scott, M.F., Pattini, L., Lupi, M., Ubezio, P., Piccotti, F., Zambelli, A., Rizzo, P., Fisher, J.N., Terao, M., Fratelli, M., Kurosaki, M., Paroni, G., Zanetti, A., Gianni, M., Bolis, M., Lupi, M., Tsykin, A., Goodall, G.J., and Garattini, E. (2015). MicroRNA networks regulated by all-

trans retinoic acid and Lapatinib control the growth, survival and motility of breast cancer cells. Oncotarget 6, 13176-13200.

Fuchs, B., Suss, R., Teuber, K., Eibisch, M. and Schiller, J. (2011). Lipid analysis by thin-layer chromatography-A review of the current state. J Chromatogr A 1218: 2754-2774.

Kraus, D., Yang, Q. and Kahn B.B. (2015) Lipid Extraction from mouse feces. Bio-protocol, 5, January 5, 2015 (http://www.bio-protocol.org/e1375).

Frezza C, Cipolat S, Scorrano L. (2007) Organelle isolation: functional mitochondria from mouse liver, muscle and cultured fibroblasts. Nat Protoc, 2:287-95.

Gianni, M., Fratelli, M., Terao, M., and Garattini, E. (2015). Cellular and molecular determinants of all-trans retinoic acid sensitivity in breast cancer: Luminal phenotype and RARalpha expression. EMBO Mol Med 7, 950-972.

Sarachana T, Hu VW (2013) Genome-wide identification of transcriptional targets of RORA reveals direct regulation of multiple genes associated with autism spectrum disorder. Mol Autism 4(1): 14.

Spinazzi M, Casarin A, Pertegato V, Salviati L, Angelini C. (2012) Assessment of mitochondrial respiratory chain enzymatic activities on tissues and cultured cells. Nat Protoc, 7: 1235-46.

Terao, M., Kurosaki, M., Barzago, M.M., Fratelli, M., Bagnati, R., Bastone, A., Giudice, C., Scanziani, E., Mancuso, A., Tiveron, C., and Garattini, E. (2009). Role of the molybdoflavoenzyme aldehyde oxidase homolog 2 in the biosynthesis of retinoic acid: generation and characterization of a knockout mouse. Mol Cell Biol 29, 357-377.

SUPPLEMENTARY DATA

Suppl. Table S1 *Genes differentially expressed in HG, WADT and liver of Aox4$^{-/-}$ mice and pathway enrichment analysis*

The table contains the list of genes significantly up- or down-regulated in the *HG, WADT* and liver of *Aox4$^{-/-}$* relative to *WT* mice. Only genes with a Log2 fold change above +0.5 and below -0.5 are listed. Each expression value corresponds to a single animal. REP = replicate value. Pathway enrichment analysis was performed on the genes differentially up- or down-regulated in *HG*, *WADT* and liver of *Aox4$^{-/-}$* relative to *WT* mice. Total = total number of genes constituting the pathway; FDR = False Discovery Rate; In Data = the value indicates the number of differentially expressed genes which are part of the pathway. The pathways of interest for the phenotype of *Aox4$^{-/-}$* animals are boxed in yellow.

Suppl. Table S2 *Metabolomics analyses performed in HGs of Aox4$^{-/-}$ and WT mice*

The table lists the metabolites whose levels are significantly different in the *HG* of *Aox4$^{-/-}$* and *WT* animals.

Suppl. Table S3 *Genes differentially expressed in HG, WADT and liver of Aox4$^{-/-}$ mice fed HFD and pathway enrichment analysis*

The table contains the list of genes significantly up- or down-regulated in *HG, WADT* and liver of *Aox4$^{-/-}$* relative to *WT* mice subjected to *HFD* for 2 months. Only genes with a Log2 fold change above +0.5 and below -0.5 are listed. Each expression value corresponds to a single animal. REP = replicate value. Pathway enrichment analysis was performed on the genes differentially up- or down-regulated in *HG*, *WADT* and liver of *Aox4$^{-/-}$* relative to *WT* mice subjected to *HFD*. Total = total number of genes constituting the pathway; FDR = False Discovery Rate; In Data = the value

indicates the number of differentially expressed genes which are part of the pathway. The pathways of interest for the phenotype of *Aox4*$^{-/-}$ animals are boxed in yellow.

Suppl. Table S4 *Rorα target genes regulated in Aox4*$^{-/-}$ *mice*

The table contains the overlaps between the list of genes significantly up- or down-regulated in our experimental conditions and the list of putative *Rorα* direct target genes (Sarachana et al., 2013). The significance of the enrichments is calculated with the hypergeometric test. A representation factor >1.0 would indicate enrichment.

Suppl. Fig. S1 *Expression of circadian rhythm genes and AOXs in HG, WADT and Liver*

(**a**) The heatmaps of the Log_2 fold changes (*Aox4*$^{-/-}$ *vs WT*) in the expression of the genes belonging to the "Circadian Rhythms" pathway observed in the indicated tissues are shown. Each box represents the mean of 4 separate *Aox4*$^{-/-}$ and *WT* animals. The genes are listed in a descending order of fold changes. (**b-d**) Expression of the *Per2*, *Dbp* and *Arntl* mRNAs was evaluated in HG (**b**), WADT (**c**) and liver (**d**) of *Aox4*$^{-/-}$ and *WT* mice by RT-PCR on the same samples used to determine the levels of the three transcripts with the gene-expression microarray platform. Each value is the mean$\pm$S.D of 4 animals *Significantly different (Student's t-test, $p<0.05$).

Suppl. Fig. S2 *Circadian oscillations of Rev-erbβ mRNA expression in HG, liver and WADT of male and female animals*

The linear graphs show the levels of the clock gene, *Rev-erbβ*, mRNA measured by PCR at the indicated zeitgebers (*ZT*) in *HD*, liver and *WADT* of female and male animals. Values are the mean$\pm$SE of 4 mice.

Suppl. Fig. S3 *Protoporphyrin IX and melatonin in the HG of Aox4*$^{-/-}$ *and WT mice*

(**a**) At the indicated zeitgebers (*ZT*), the levels of protoporphyrin IX in *Aox4*$^{-/-}$ and *WT HGs* were determined. Protoporphyrin IX was extracted from homogenates with acidic ethanol and fluorescence intensity was measured (Excitation: 400 nm; Emission: 605 nm) using 1 µM mesporphyrin IX as a standard. Each value is the mean$\pm$SD of 5 distinct mice. White boxes = light-phase; Black boxes = dark-phase. *Significantly different according to the Student's t-test ($p<0.05$). **Significantly different according to the Student's t-test ($p<0.01$). (**b**) The panel illustrates the mass-spectra of melatonin and the corresponding external standard (Kynurenin-D4) which were determined in the indicated samples.

Suppl. Fig. S4 *OPLS-DA analysis of metabolomics data in HG of Aox4*$^{-/-}$ and *WT* animals.

(**a**) OPLS-DA score plot showing classes separated according to their metabolic signature (POSITIVE ION mode), where classes correspond to *Aox4*$^{-/-}$ and *WT*. (**b**) S-plot highlighting which ions of interest (POSITIVE ION mode) are up or down regulated in their respective group. (**c**) OPLS-DA score plot showing classes separated according to their metabolic signature (NEGATIVE ION mode), where classes correspond to *Aox4*$^{-/-}$ and *WT*. (**d**) S-plot highlighting which ions of interest (NEGATIVE ION mode) are up or down regulated in their respective group.

Suppl. Fig. S5 *Serotonin and melatonin biosynthetic pathway*

The boxed compounds indicate the metabolites of the serotonin/melatonin pathway whose levels are significantly different in *Aox4*$^{-/-}$ and *WT* mice, as assessed by metabolomics.

Suppl. Fig. S6 *Tryptophan and 5HIAA are transformed into mono-hydroxylated products by AOX4 and AOX3*

(**a**) Purified AOX4 and AOX3 (0.3 and 0.5µg) were incubated with tryptophan (10µM), 5HIAA (1mM) or serotonin (1mM). Enzymatic activity was measured with a peroxidase coupled fluorescence assay. The negative controls shown were run in the absence of the two enzymes, although similar results were obtained after boiling the enzymes for 10 minutes prior to the assay. In the leftmost panel illustrating the results obtained with serotonin, the internal positive control of the experiment is represented by 5HIAA, as indicated. (**b**) The panels illustrate the LC-MS/MS results obtained after incubation of 5HIAA with no enzyme (1), 25 µg AOX4 (2) and 75 µg AOX3 (3) in 200 µl 20 mM ammonium acetate (pH 7.5) at 37°C for 5 minutes. Upper: The HPLC separation chromatogram is presented. Lower: The fragmentation profiles of tryptophan and mono-hydroxylated products are shown as indicated. (**c**) The panels show the LC-MS/MS results obtained after incubation of tryptophan with no enzyme (4), purified AOX4 (5) and purified AOX3

(6). Panel (7) shows the results obtained with the 5-OH tryptophan standard. The upper and lower sections of each panel are as in (**b**).

Suppl. Fig. S7 *Mitochondrial respiratory chain enzymatic activities in HG of Aox4$^{-/-}$ and WT mice*

*HG*s of *Aox4*$^{-/-}$ and *WT* mice kept under standard conditions were used to isolate mitochondria. Crude mitochondrial fractions were obtained from individual animals (4 mice/experimental group) as detailed in SUPPLEMENTARY METHODS and the indicated mitochondrial enzymatic activities were measured. Values are the mean±SE of at least 4 samples. * Significantly different (Student's t-test, $p<0.05$).

Suppl. Fig. S8 *Body weight of female and male WT* and *Aox4*$^{-/-}$

Female or male *WT* and *Aox4*$^{-/-}$ mice were subjected to normal (*ND*) and high fat (*HFD*) for 110 or 60 days. The panels show the body weight curves of female and male *WT* and *Aox4*$^{-/-}$ mice. Values are the mean±SD of 10 mice. ** Significantly different (Student's t-test, $p<0.01$).

Suppl. Fig. S9 *Analysis of faeces and urines in Aox4$^{-/-}$ and WT mice*

(**a**) and (**b**) Eight animals/experimental group were housed in four metabolic cages (2 mice/cage) and maintained under standard conditions for a period of 24 hours. Faeces (**a**) and urines (**b**) were collected and the corresponding amounts or volumes measured. The results are representative of 3 independent experiments and do not show significant differences between *Aox4*$^{-/-}$ and *WT* female or male animals following t-test. The results are the mean±SD. (**c**) Left: Twelve *Aox4*$^{-/-}$ or *WT* animals/experimental group were fed *ND* or *HFD*, as indicated, for 15 days. Faeces from single cages housing 3 animals/cage were collected during the last 3 days and total lipids were extracted with chloroform/methanol. The content of total lipids in the faeces (mg/gram of faeces) is illustrated and each value is the mean+SE of 4 separate cages/experimental group. ** Significantly different (Student's t-test, $p<0.01$). Right: The panel illustrates the qualitative lipid composition of

the faecal chloroform/methanol extracts of *Aox4*$^{-/-}$ or *WT* animals subjected to *ND* or *HFD*, as assessed by thin layer chromatography. The migration of the following lipid standards is indicated on the left: 1 = cholesterol esters; 2 = triglycerides; 3 = diglycerides; 4 = free fatty acids; 5 = cholesterol; 6 = phospholipids.

Suppl. Fig. S10 *Expression of BADT marker mRNAs in WADT of Aox4*$^{-/-}$ *and WT mice*

Female *WT* and *Aox4*$^{-/-}$ mice were subjected to *ND* and *HFD* for 110 days. The graphs illustrate the expression of the indicated mRNAs in *WADT*, as determined by microarray analysis. Values are the mean±SE of 4 mice. **Significantly different (Student's t-test, p<0.01).

Suppl. Fig. S11 *Mitochondrial respiratory chain enzymatic activities in WADT of Aox4*$^{-/-}$ *and WT mice*

Aox4$^{-/-}$ and *WT* mice were subjected to ND and HFD for 110 days. Crude mitochondrial fractions were isolated from individual animals as detailed in SUPPLEMENTARY METHODS and the indicated mitochondrial enzymatic activities were measured. Values are the mean±SE of at least 4 mice. * Significantly different (Student's t-test, p<0.05); ** Significantly different (Student's t-test, p<0.01).

Suppl. Fig. S12 *Effect of Insulin on the phosphorylation/activation of the Insulin Receptor and the downstream AKT kinase in WADT, liver and muscles of Aox4*$^{-/-}$ *and WT mice*

Aox4$^{-/-}$ and *WT* mice were starved overnight. The next morning, 6 animals/experimental group were treated with vehicle (DMSO) or 750 milliunits/kg of insulin intraperitoneally. Total extracts of inguinal WADT, liver and a mixture of soleus and gastrocnemium muscles were subjected to Western Blot analysis for the indicated proteins. IR = Insulin receptor; AKT = AKT serine/threonine kinase 1. Tubulin was used as a loading control. Each lane represents an individual animal. The column graphs indicate the densitometric analyses of the phosphorylated IR

and AKT bands, as indicated. The values are calculated as ratio of the densitometric volumes of the phosphorylated-IR and total-IR (IRp/IR) or the phosphorylated-AKT and total-AKT (AKTp/AKT) bands. Each value is the mean$\pm$S.E. of 3 individual pooled extracts (2 individual animals/pool).

Suppl. Fig. S13 *Body temperature of female and male Aox4$^{-/-}$ and WT mice*

The graphs show the rectal body temperatures in female and male *Aox4*$^{-/-}$ and *WT* animals measured at different zeitgebers. The first points (ZT=4) are repeated at the end of the graph to better represent the circadian rhythm oscillations. Each value is the mean$\pm$SE of 45-55 measurements over a period of 12 weeks in 8-10 mice per group. White boxes=light on; Black boxes=light off.

Suppl. Fig. S14 *UCP-1 activity in BADT, abdominal fat volume and total body weight of Aox4$^{-/-}$ and WT mice kept in thermo-neutral conditions*

Female *Aox4*$^{-/-}$ and *WT mice* normally kept at 22°C in standard animal house conditions were maintained for 30 days in thermoneutral conditions (30°C). (**a**) Intra-scapular brown adipose tissue (BADT) was isolated from each animal. Total RNA was extracted and the levels of UCP-1 mRNA were determined by a specific Taqman assay. Values are the mean$\pm$SE of 4 mice. ** Significantly different (Student's t-test, p<0.01). (**b**) The abdominal fat volume (MRI analysis) was determined as detailed for Fig. 6a and 6b before switching the animals from 22°C to 30°C (time 0) and after 30 days at 30°C (time 30). ** Significantly different (Student's t-test, p<0.01). (**c**) The panel illustrates the total body weight determined at the indicated times following switching from 22°C (time 0) to 30°C. Values are the mean$\pm$SE of 8 animals.

Suppl. Fig. S15 *Serum ATRA levels of Aox4$^{-/-}$ and WT mice*

The graphs show the serum levels of ATRA measured in *Aox4*$^{-/-}$ and *WT* animals by mass-spectrometry, using Acitretin as an internal standard. The first points (ZT=4) are repeated at the end of the graph to better represent the circadian rhythm oscillations. Each value is the mean$\pm$SE of

10 distinct mice. White boxes=light on; Black boxes=light off. *Significantly different according to the Student's t-test ($p<0.05$).

Suppl. Fig. S16 *Map of the "Development_Insulin, IGF1 and TNFα in brown adipocyte differentiation" pathway*

The figure summarizes the changes (*Aox4*$^{-/-}$ *vs WT*) in the expression of the genes belonging to the Metacore "Development_Insulin, IGF1 and TNFα in brown adipocyte differentiation" pathway map animals in the three tissues considered. The map is based on the results obtained with the whole-genome gene expression microarrays. Only significant changes are indicated ($p<0.05$, fold change >0.6). 1 = HG of animals fed ND; 2 = HG of animals fed HFD; 3 = Liver of animals fed ND; 4 = Liver of animals fed HFD; 5 = WADT of animals fed ND; 6 = WADT of animals fed HFD

Suppl. Fig. S17 *Survival curves of Aox4*$^{-/-}$ *and WT mice*

Kaplan-Meier survival curves of female and male *Aox4*$^{-/-}$ *and WT* animals. Censored events (vertical lines) indicate animals which were sacrificed for independent reasons. The p-values are the results of COX proportional hazard analysis.

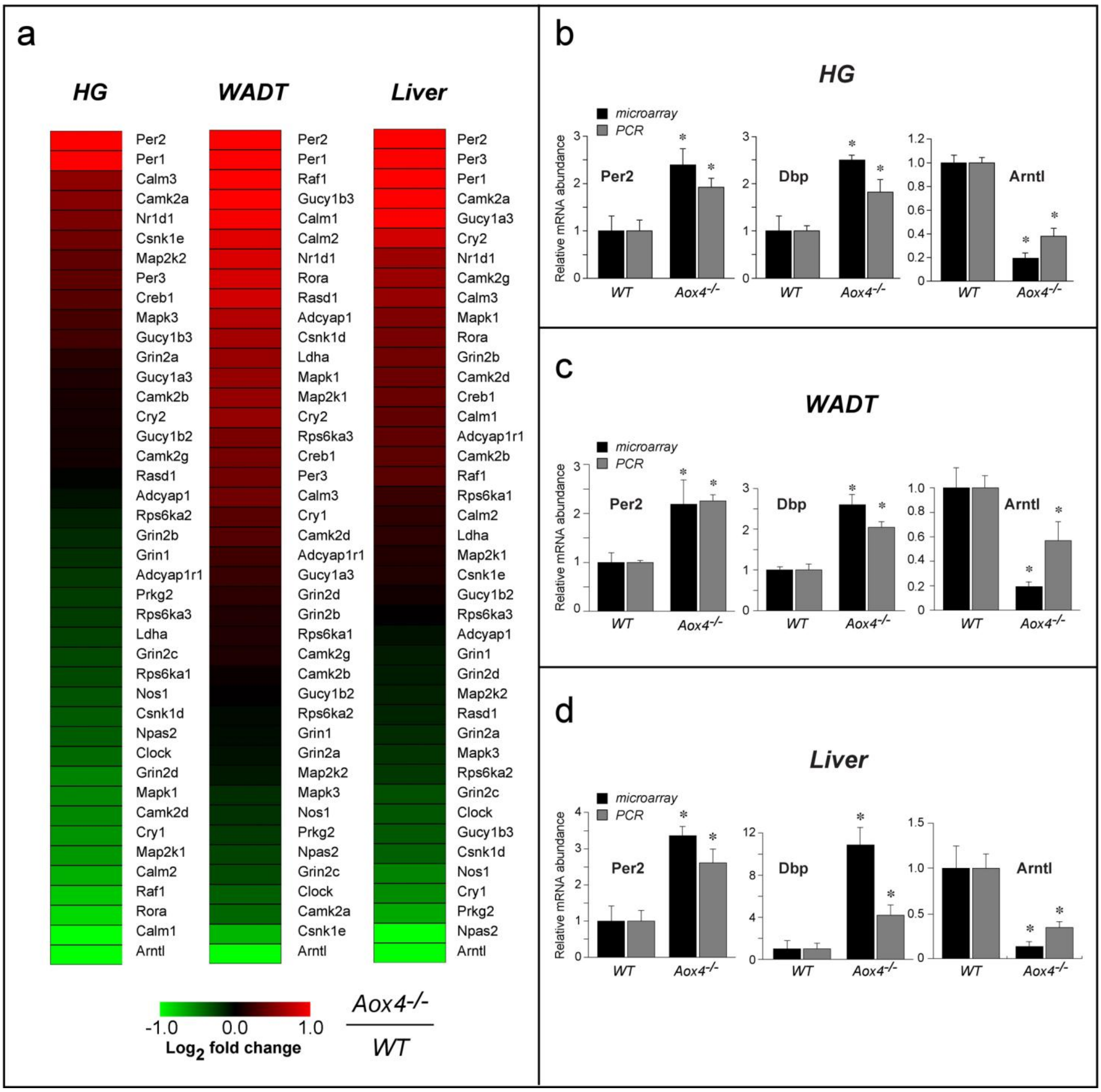
a
HG
WADT
Liver
Per2
Per1
Calm3
Camk2a
Nr1d1
Csnk1e
Map2k2
Per3
Creb1
Mapk3
Gucy1b3
Grin2a
Gucy1a3
Camk2b
Cry2
Gucy1b2
Camk2g
Rasd1
Adcyap1
Rps6ka2
Grin2b
Grin1
Adcyap1r1
Prkg2
Rps6ka3
Ldha
Grin2c
Rps6ka1
Nos1
Csnk1d
Npas2
Clock
Grin2d
Mapk1
Camk2d
Cry1
Map2k1
Calm2
Raf1
Rora
Calm1
Arntl
-1.0 0.0 1.0
Log2 fold change
Aox4-/- / WT
b
HG
c
WADT
d
Liver
microarray
PCR
Relative mRNA abundance
Per2
Dbp
Arntl
WT
Aox4-/-

Suppl. Fig. S1

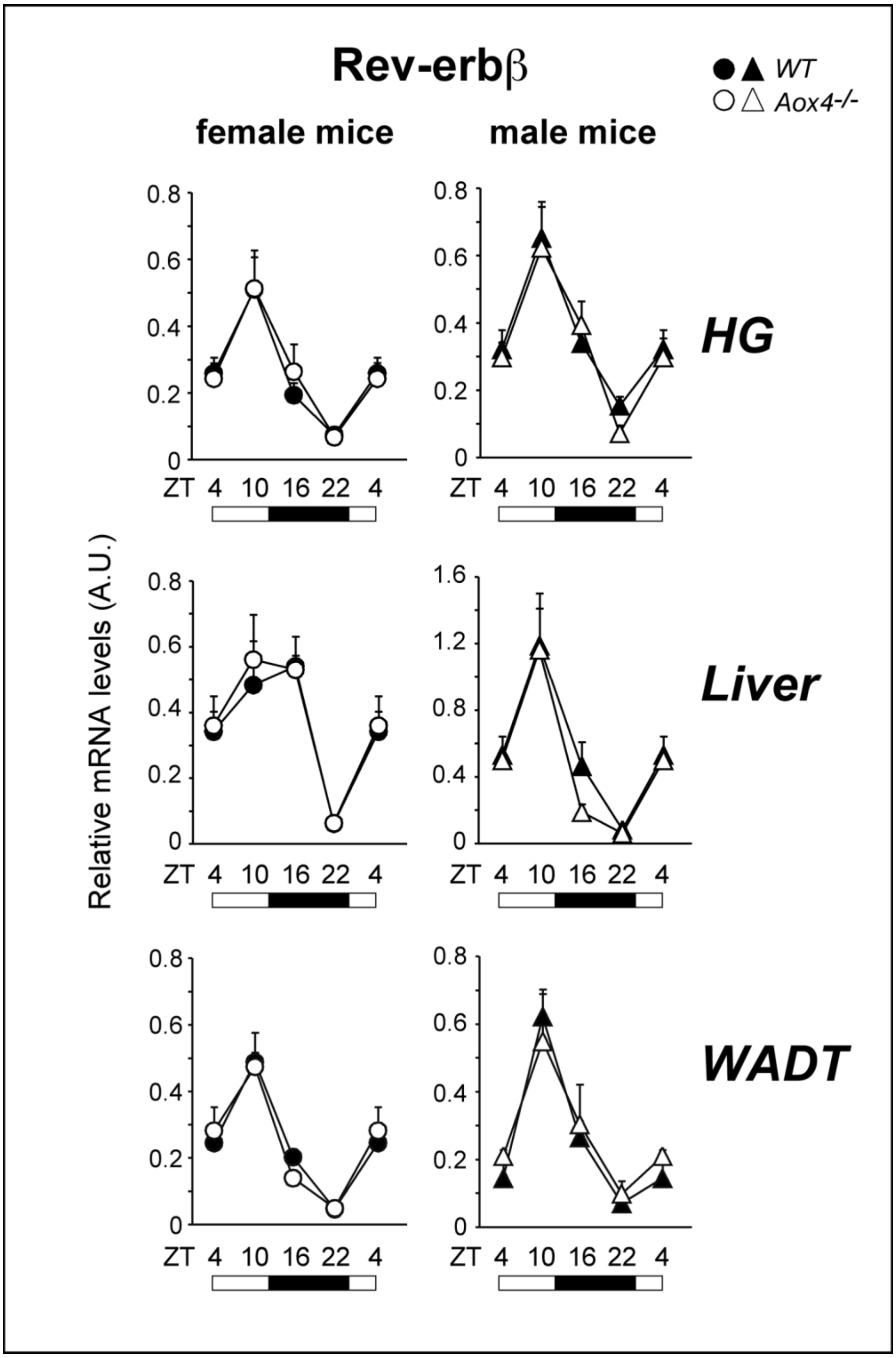

Rev-erbβ
WT
Aox4-/-
female mice
male mice
HG
Liver
WADT
Relative mRNA levels (A.U.)
ZT 4 10 16 22 4

Suppl. Fig. S2

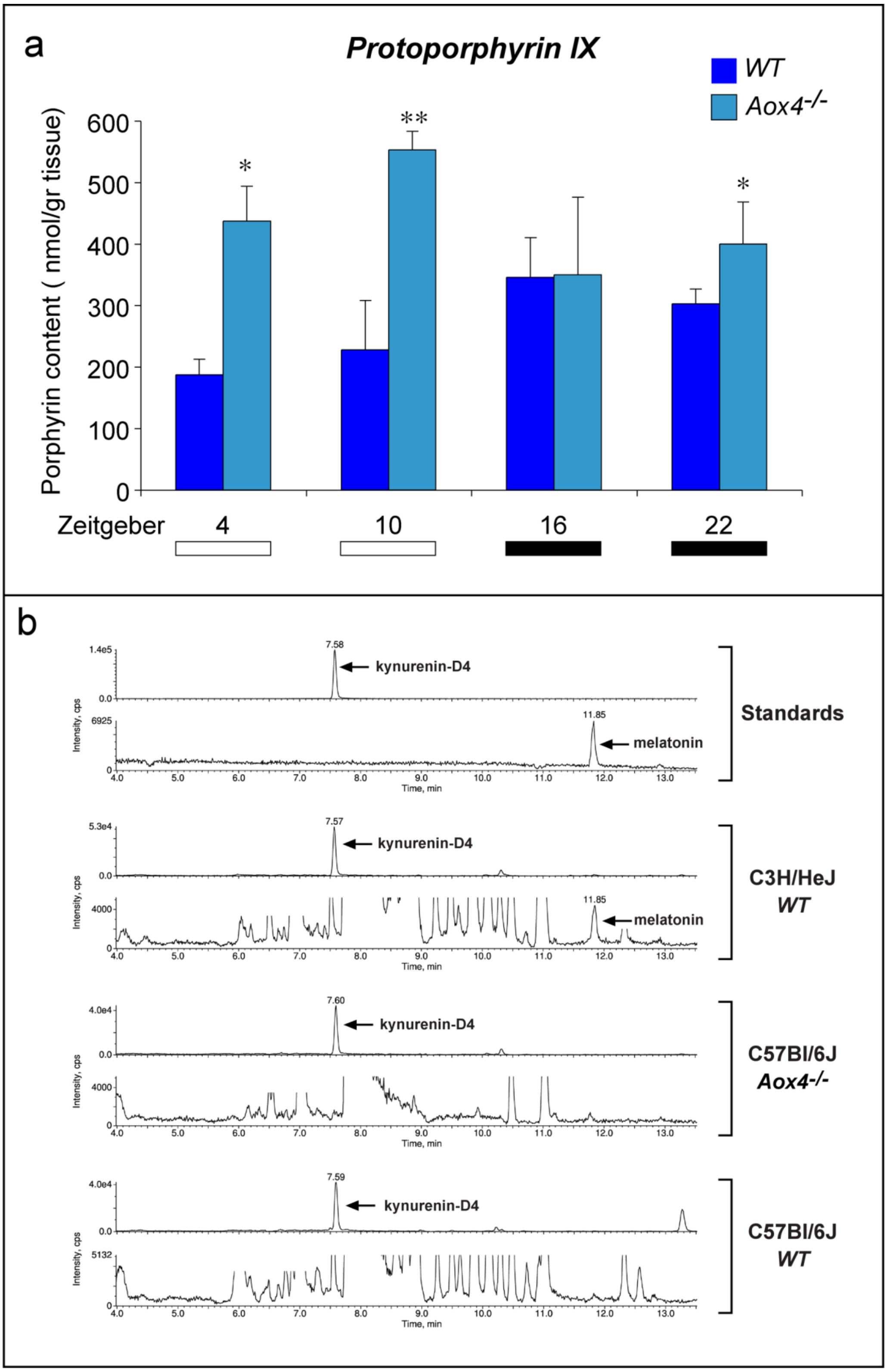
a
Protoporphyrin IX
WT
Aox4-/-
Porphyrin content (nmol/gr tissue)
600
500
400
300
200
100
0
*
**
*
Zeitgeber
4
10
16
22
b
kynurenin-D4
melatonin
Standards
C3H/HeJ
WT
C57Bl/6J
Aox4-/-
C57Bl/6J
WT
Intensity, cps
Time, min

Suppl. Fig. S3

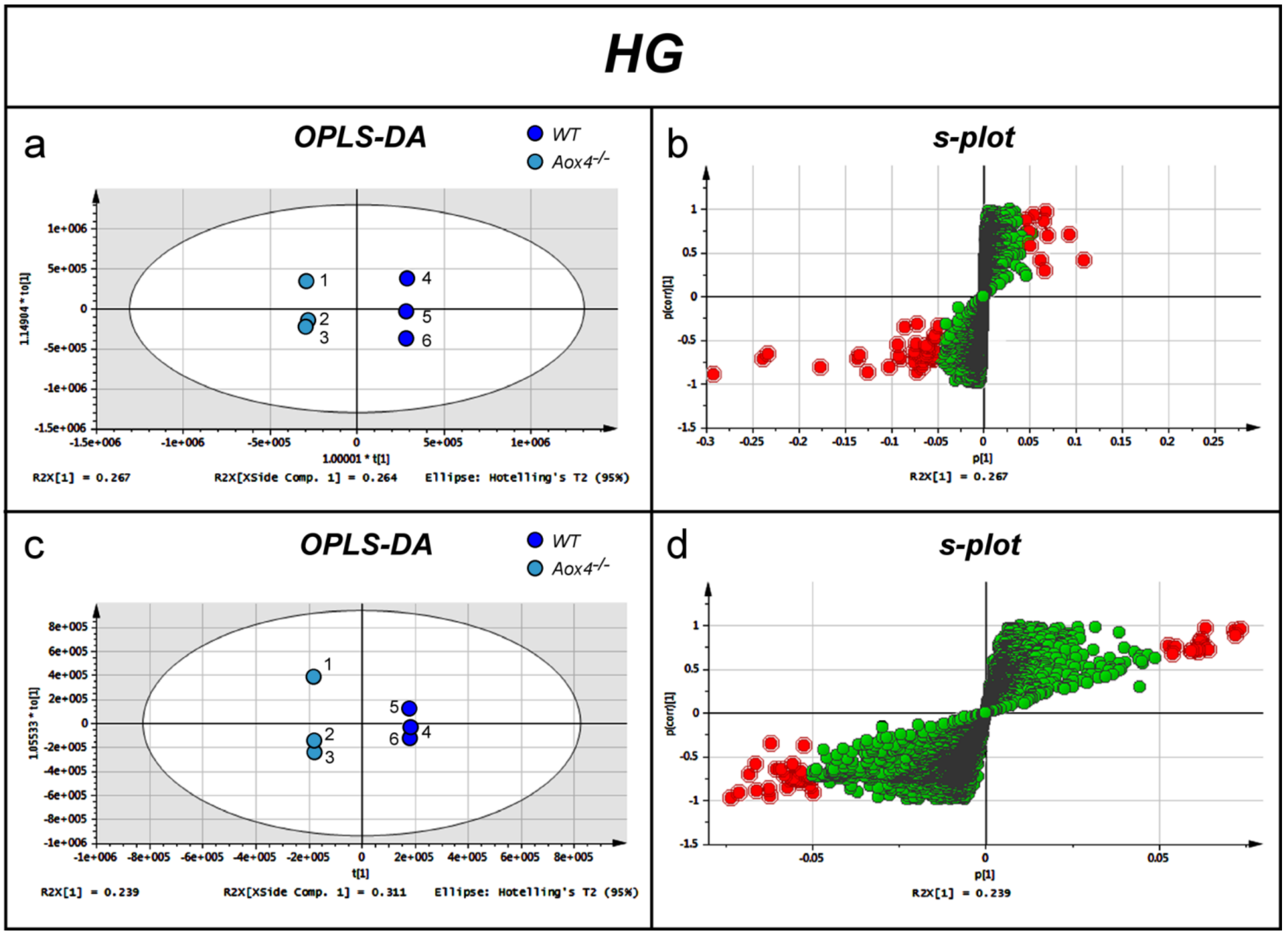
HG
a
OPLS-DA
WT
Aox4-/-
b
s-plot
c
OPLS-DA
WT
Aox4-/-
d
s-plot
R2X[1] = 0.267
R2X[XSide Comp. 1] = 0.264
Ellipse: Hotelling's T2 (95%)
R2X[1] = 0.239
R2X[XSide Comp. 1] = 0.311

Suppl. Fig. S4

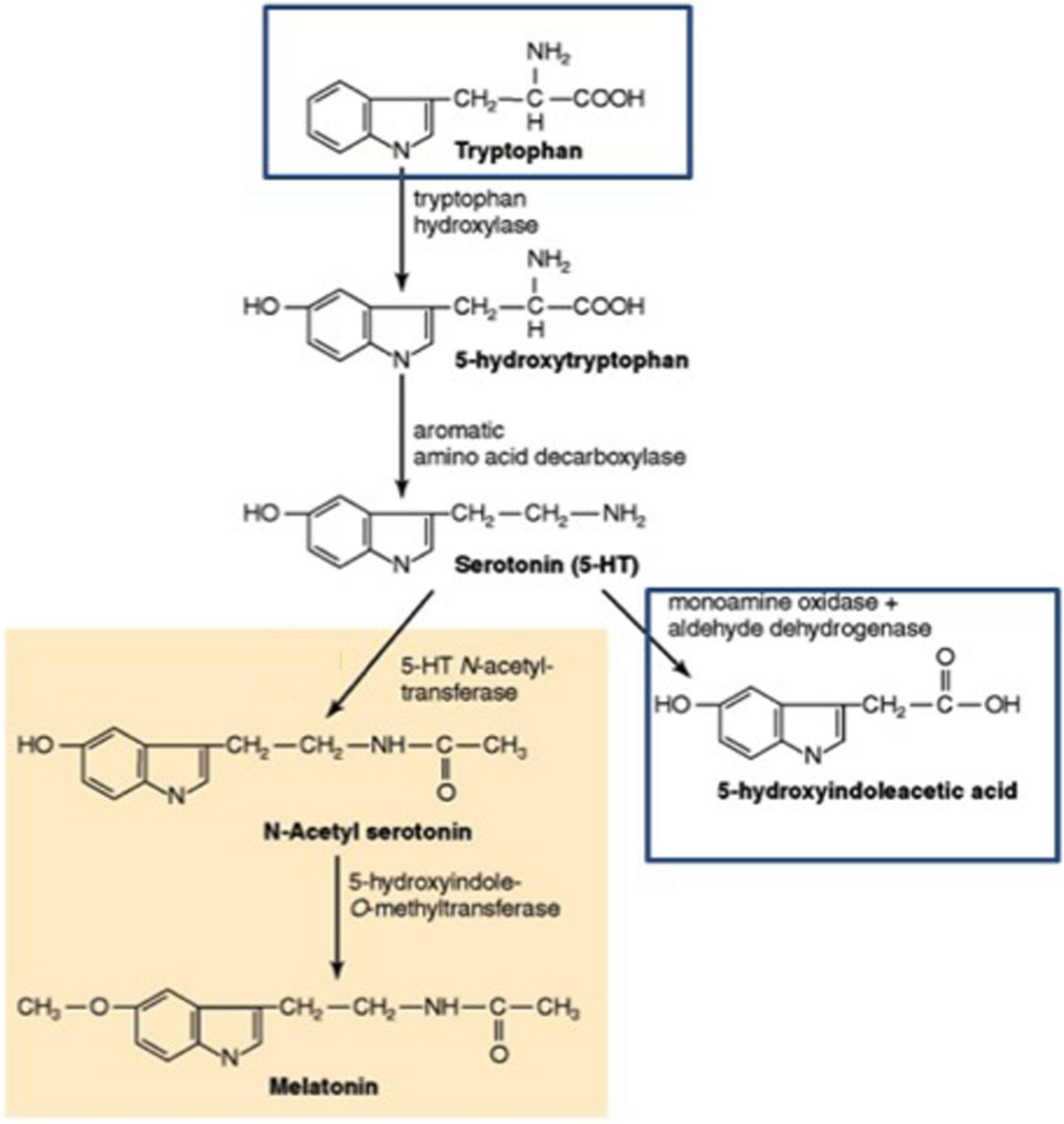

NH2
CH2—C—COOH
H
N
Tryptophan
tryptophan
hydroxylase
HO
5-hydroxytryptophan
aromatic
amino acid decarboxylase
CH2—CH2—NH2
Serotonin (5-HT)
monoamine oxidase +
aldehyde dehydrogenase
CH2—C—OH
5-hydroxyindoleacetic acid
5-HT N-acetyl-
transferase
CH2—CH2—NH—C—CH3
N-Acetyl serotonin
5-hydroxyindole-
O-methyltransferase
CH3—O
Melatonin

Suppl. Fig. S5

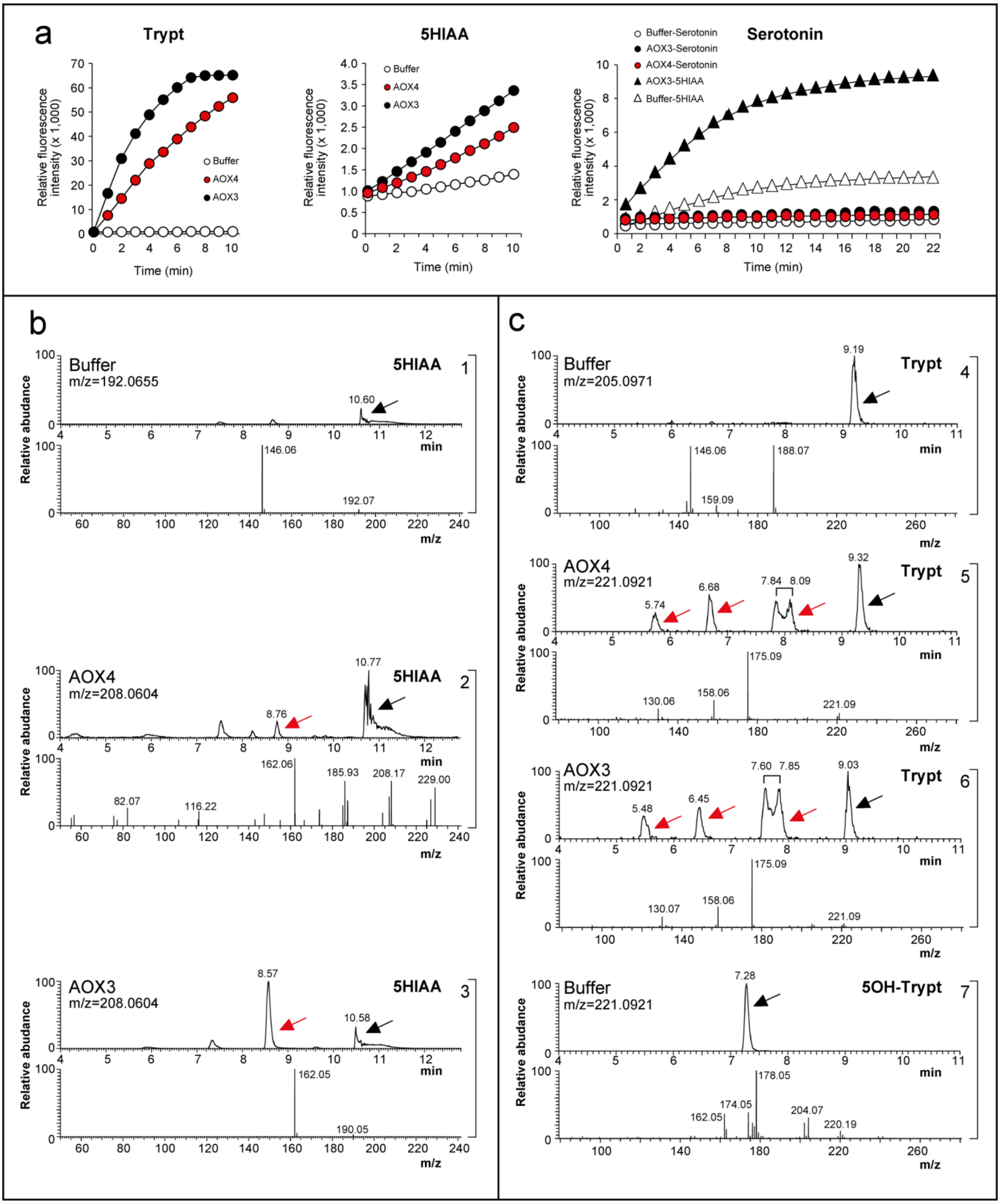
a
Trypt
Relative fluorescence intensity (x 1,000)
Time (min)
Buffer
AOX4
AOX3
5HIAA
Serotonin
Buffer-Serotonin
AOX3-Serotonin
AOX4-Serotonin
AOX3-5HIAA
Buffer-5HIAA
b
c
Relative abudance
Buffer
m/z=192.0655
5HIAA
AOX4
m/z=208.0604
AOX3
m/z=205.0971
m/z=221.0921
Trypt
5OH-Trypt
min
m/z

Suppl. Fig. S6

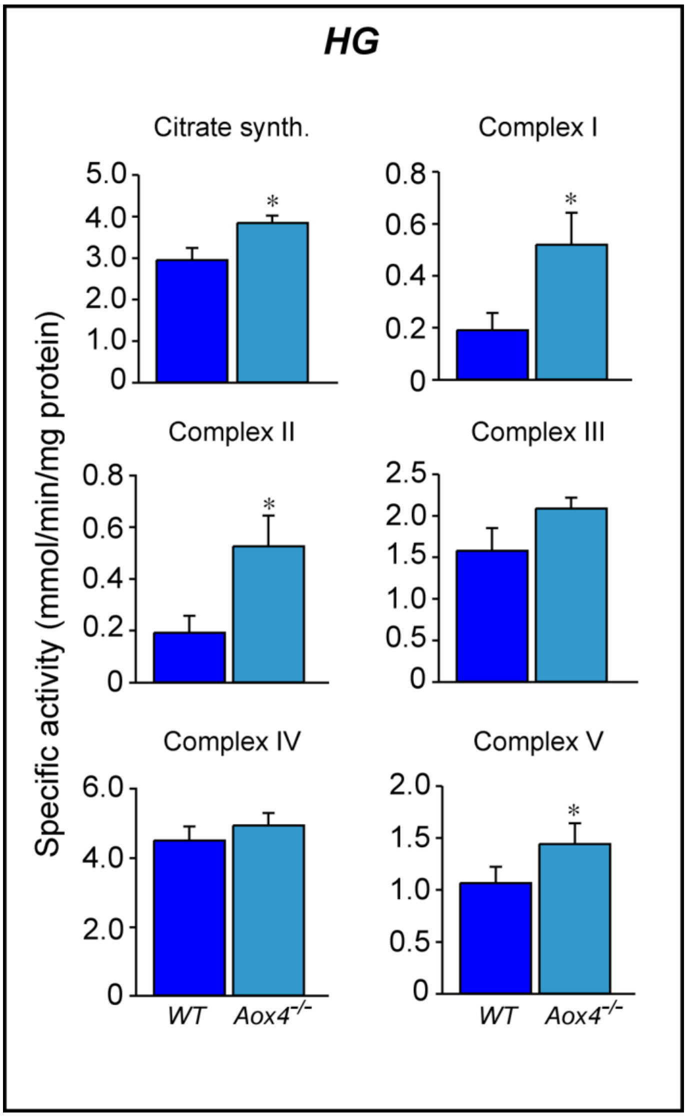
HG
Citrate synth.
Complex I
Complex II
Complex III
Complex IV
Complex V
Specific activity (mmol/min/mg protein)
5.0
4.0
3.0
2.0
1.0
0
0.8
0.6
0.4
0.2
0
2.5
2.0
1.5
1.0
0.5
6.0
*
WT
Aox4-/-

Suppl. Fig. S7

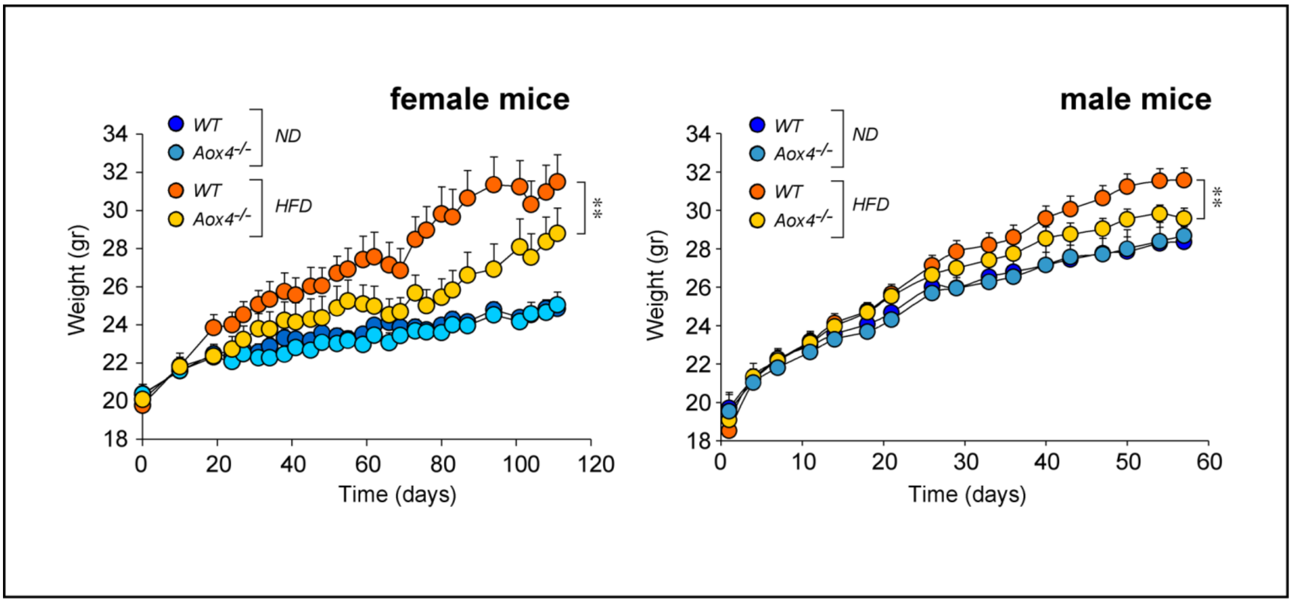
female mice
male mice
WT
Aox4-/-
ND
WT
Aox4-/-
HFD
Weight (gr)
Time (days)
**

Suppl. Fig. S8

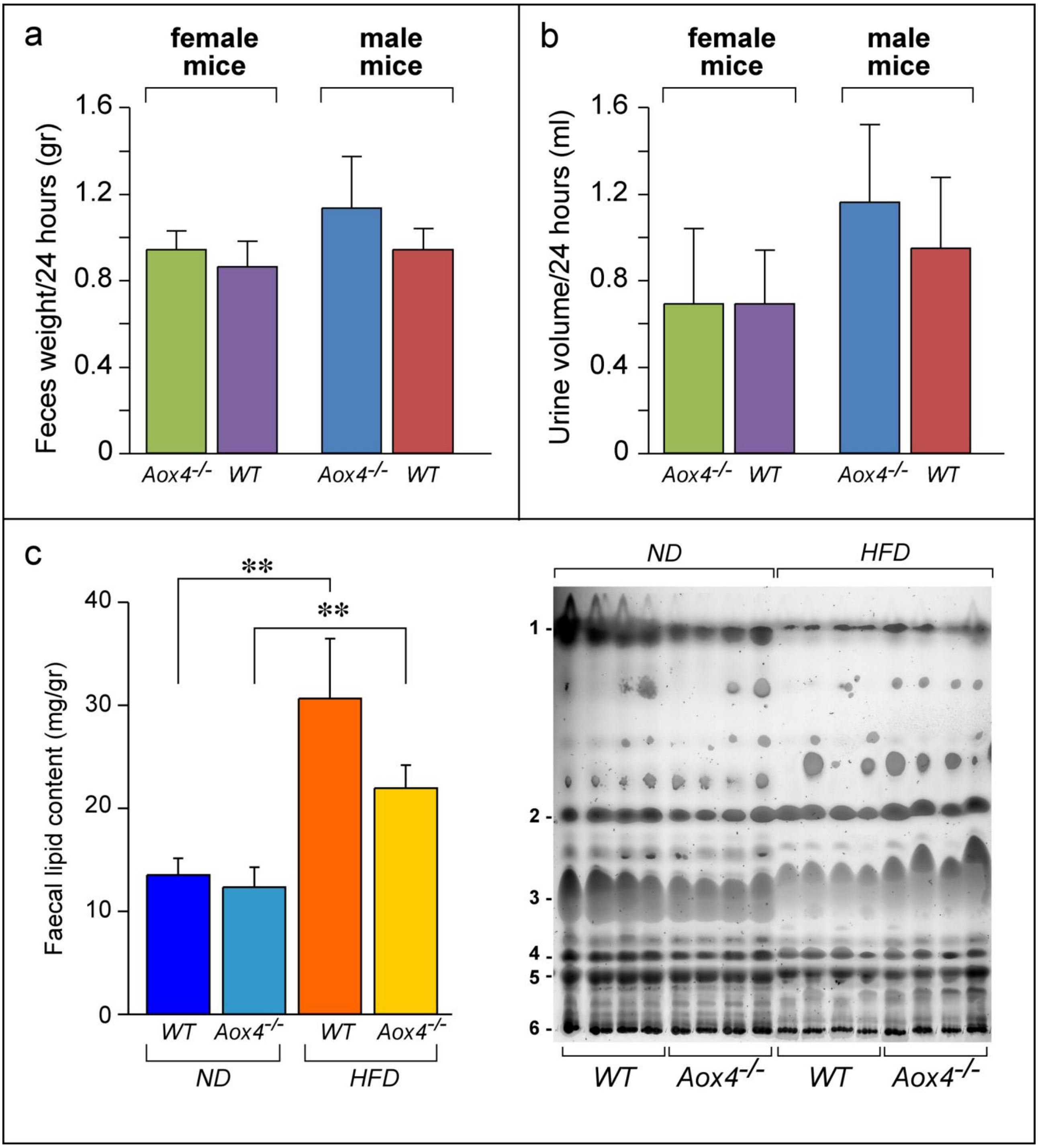
a
female mice
male mice
Feces weight/24 hours (gr)
1.6
1.2
0.8
0.4
0
Aox4-/-
WT
Aox4-/-
WT
b
female mice
male mice
Urine volume/24 hours (ml)
1.6
1.2
0.8
0.4
0
Aox4-/-
WT
Aox4-/-
WT
c
**
**
Faecal lipid content (mg/gr)
40
30
20
10
0
WT
Aox4-/-
WT
Aox4-/-
ND
HFD
ND
HFD
1
2
3
4
5
6
WT
Aox4-/-
WT
Aox4-/-

Suppl. Fig. S9

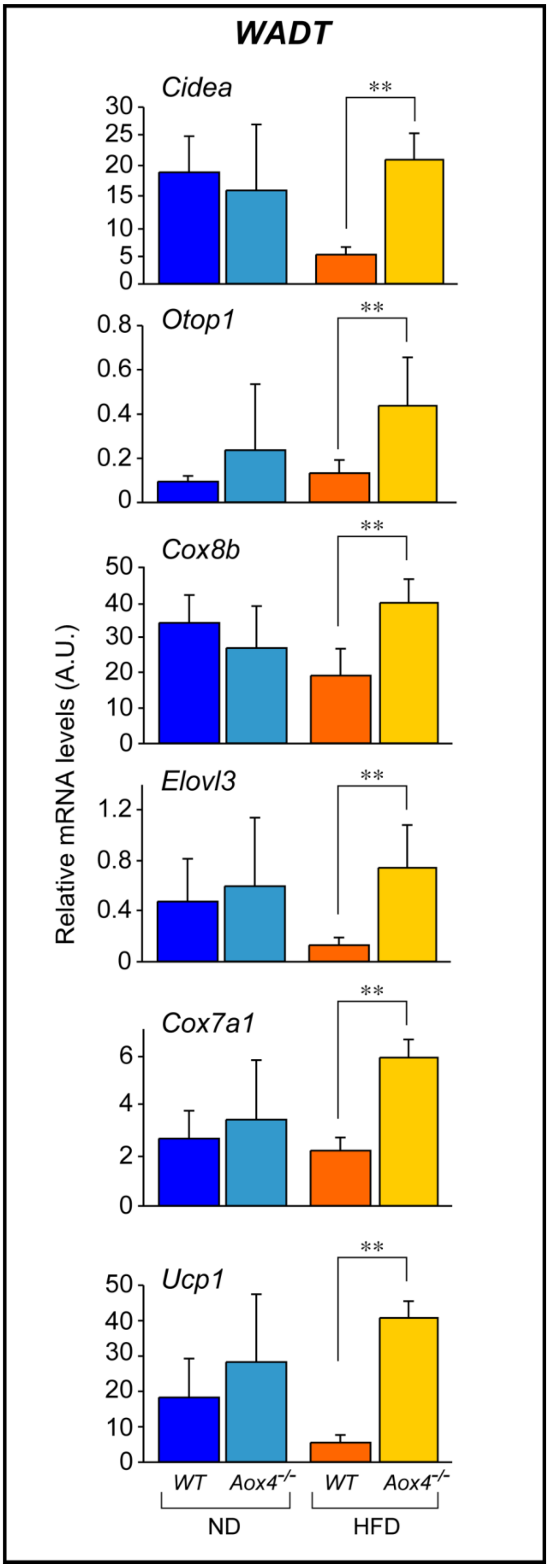

Suppl. Fig. S10

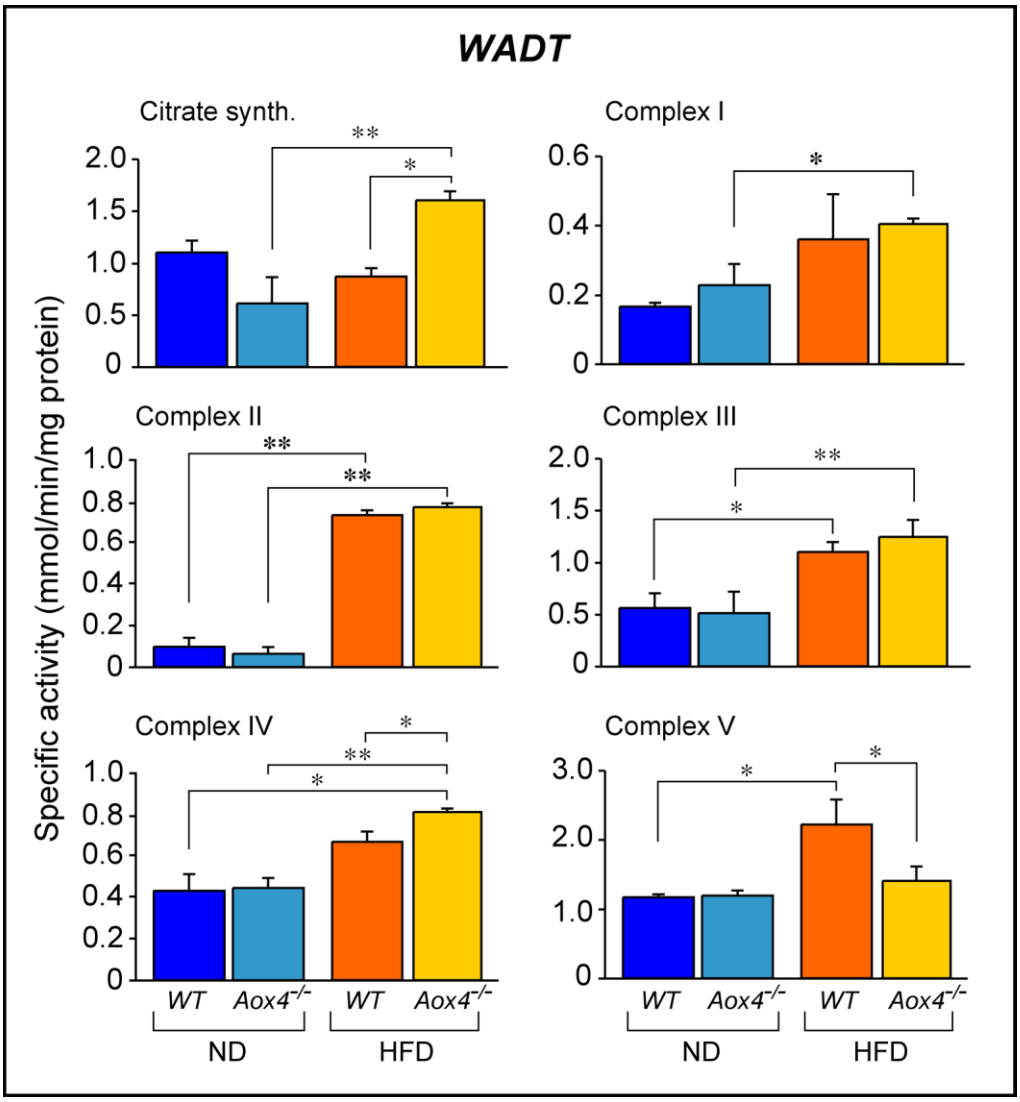

Suppl. Fig. S11

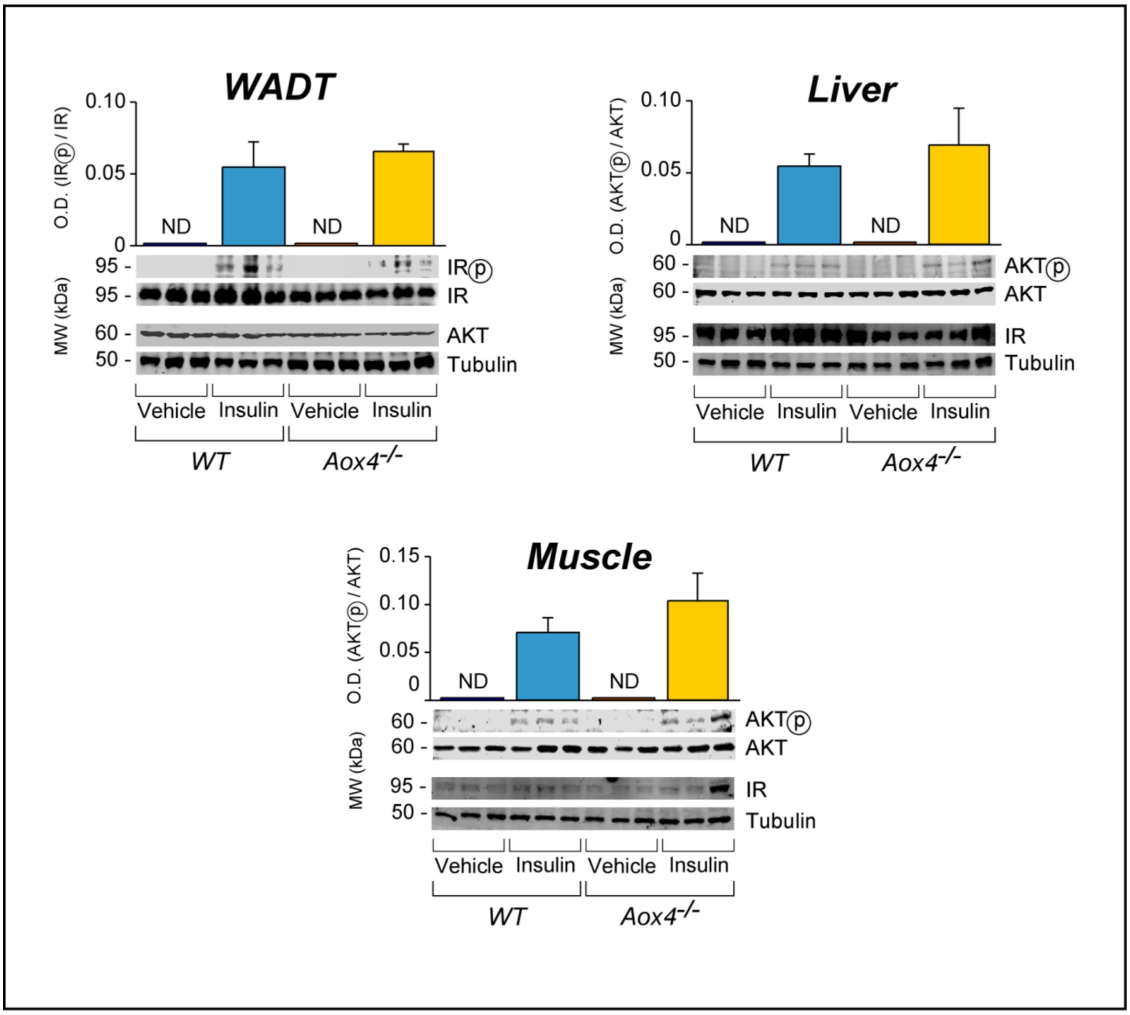

Suppl. Fig. S12

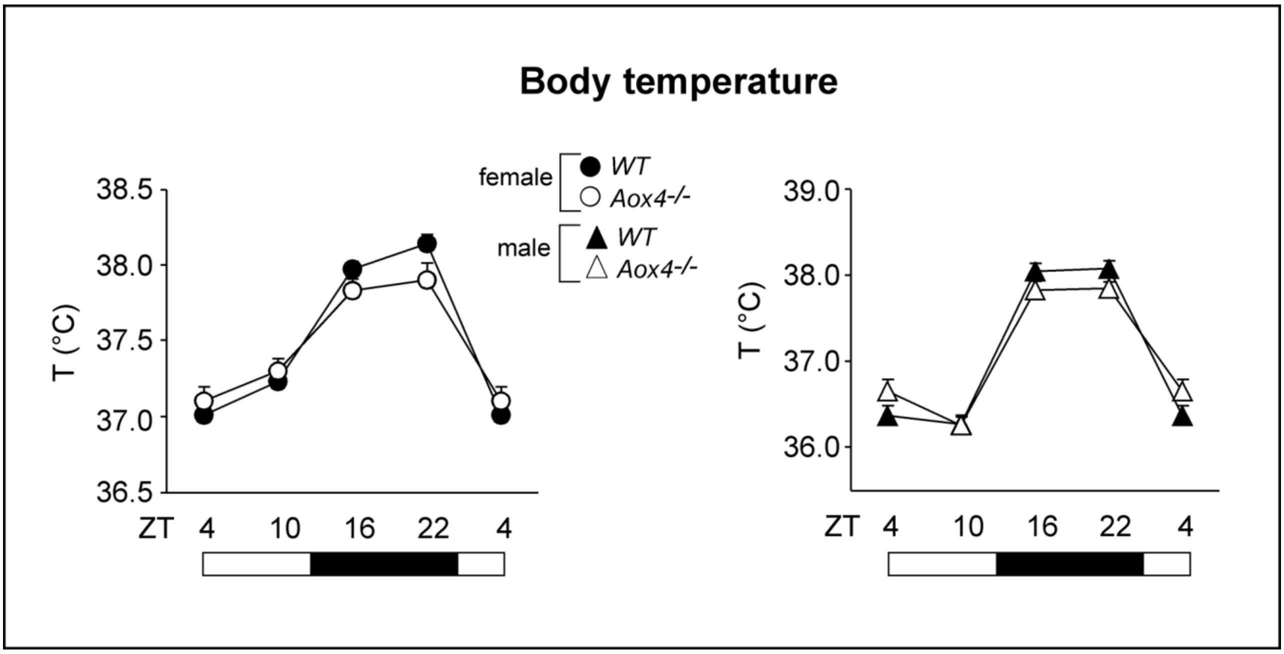

Suppl. Fig. S13

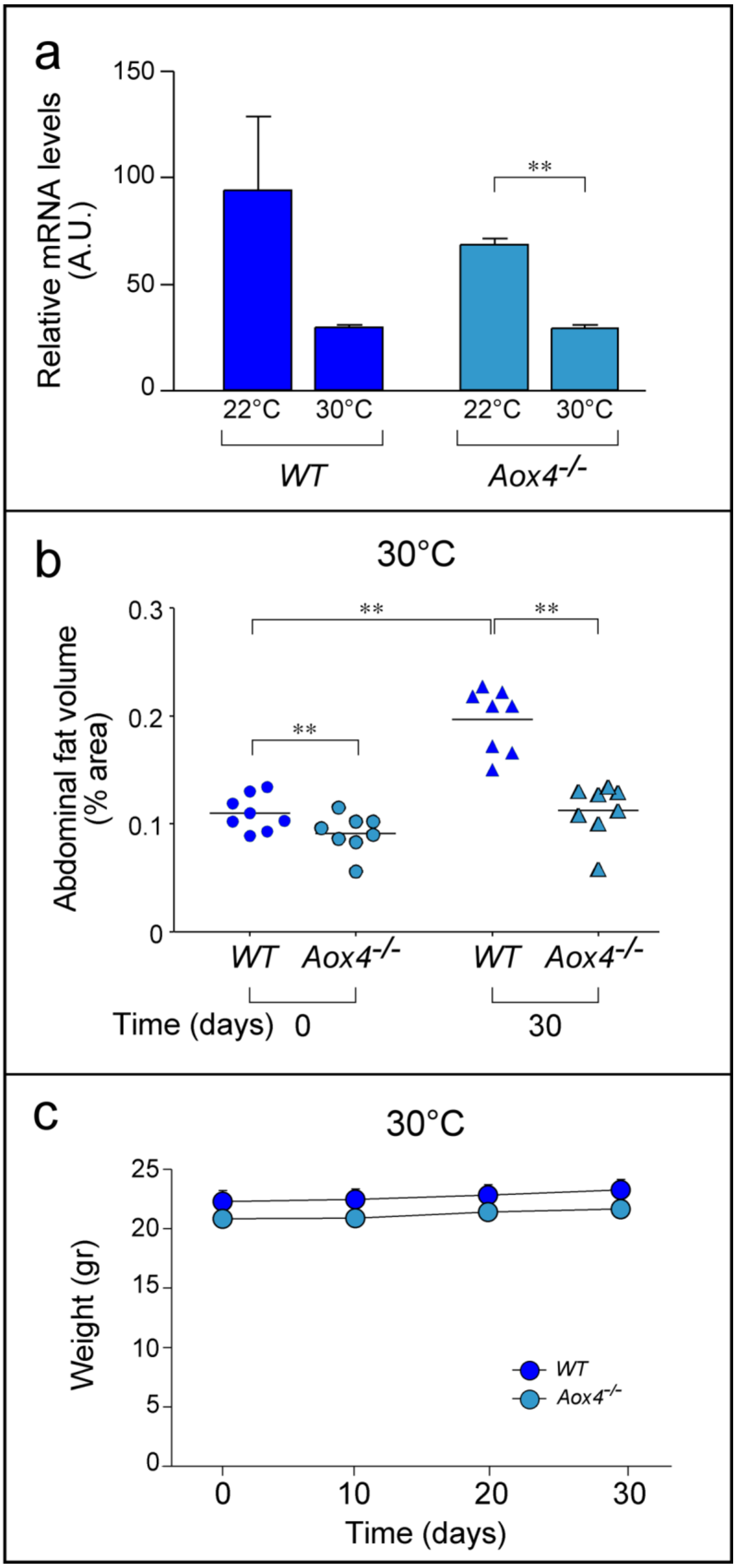

Suppl. Fig. S14

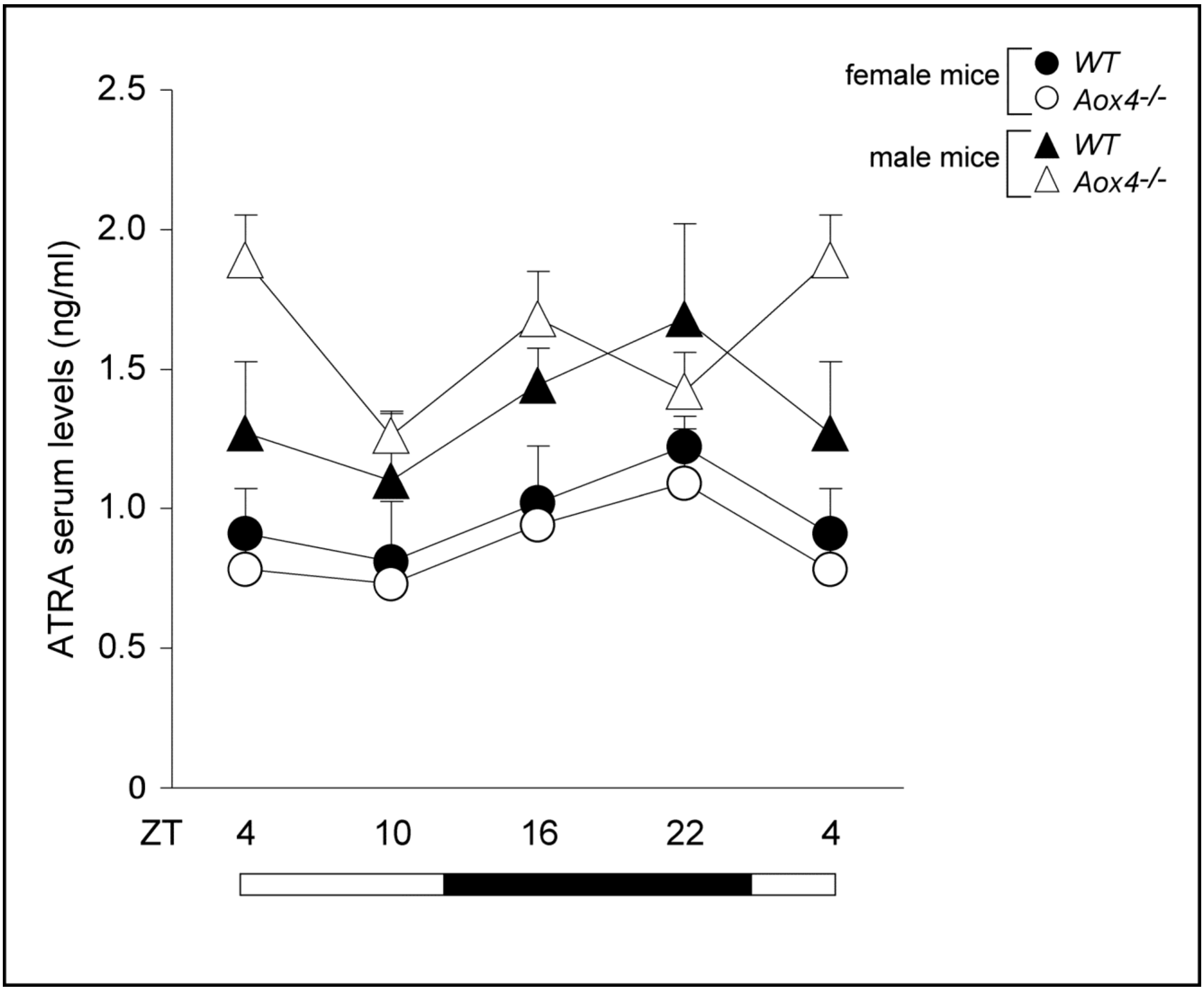

Suppl. Fig. S15

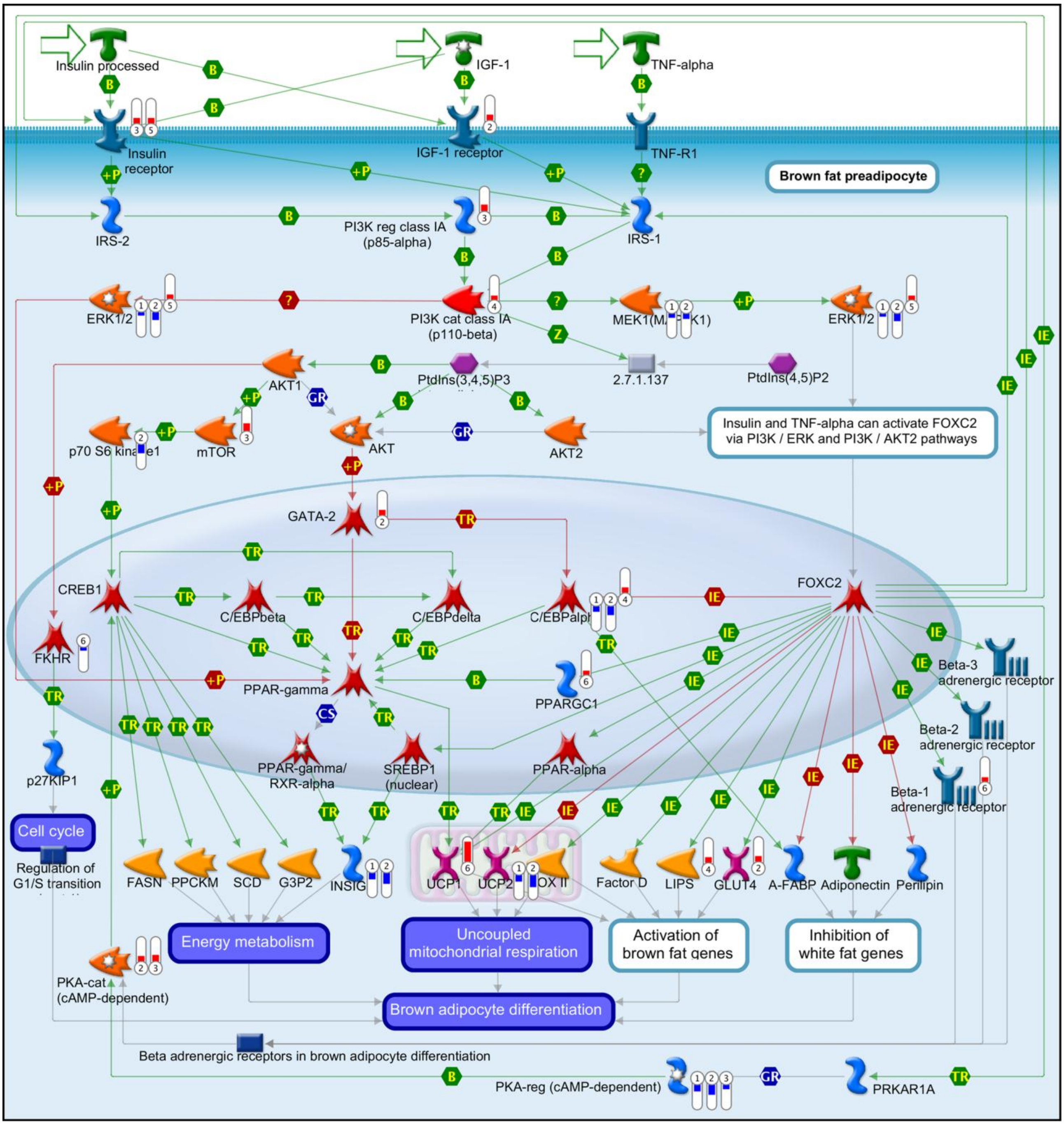

Suppl. Fig. S16

Legend to Suppl. Fig. S16

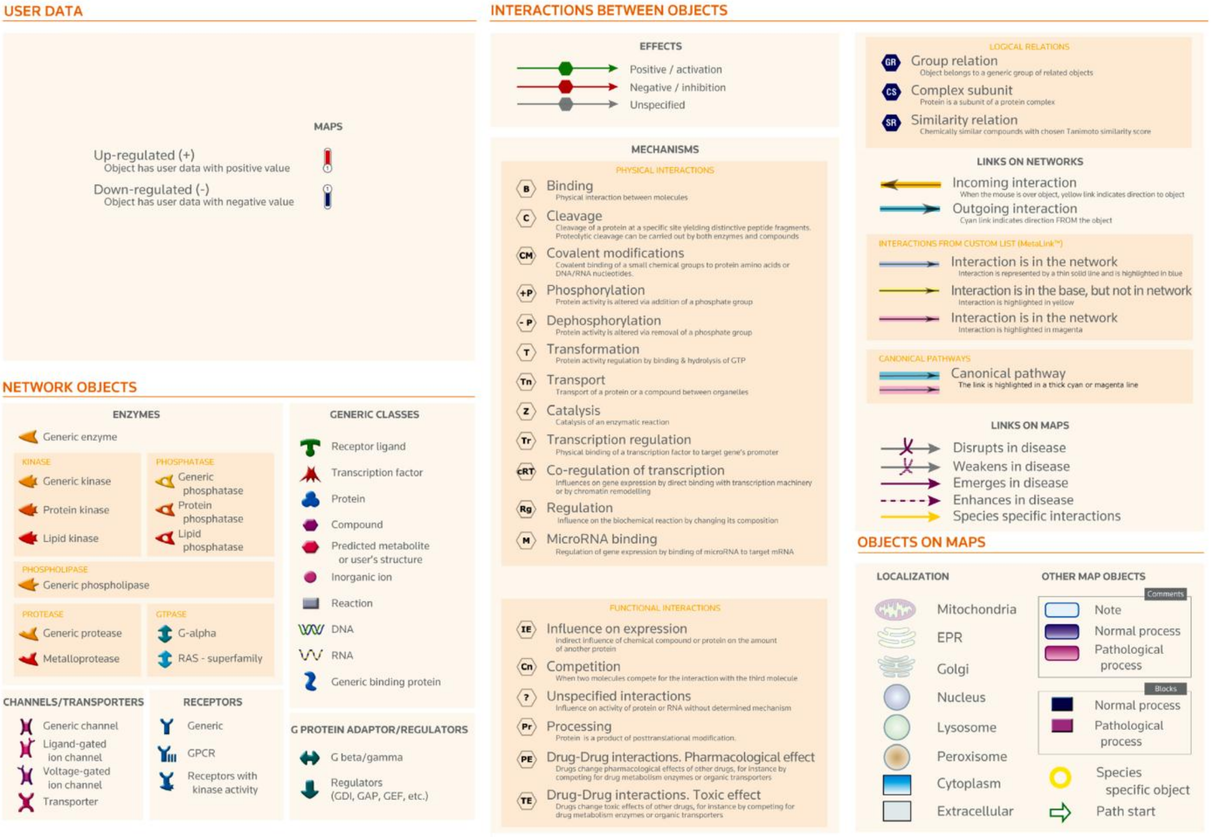

Suppl. Fig. S16 continued

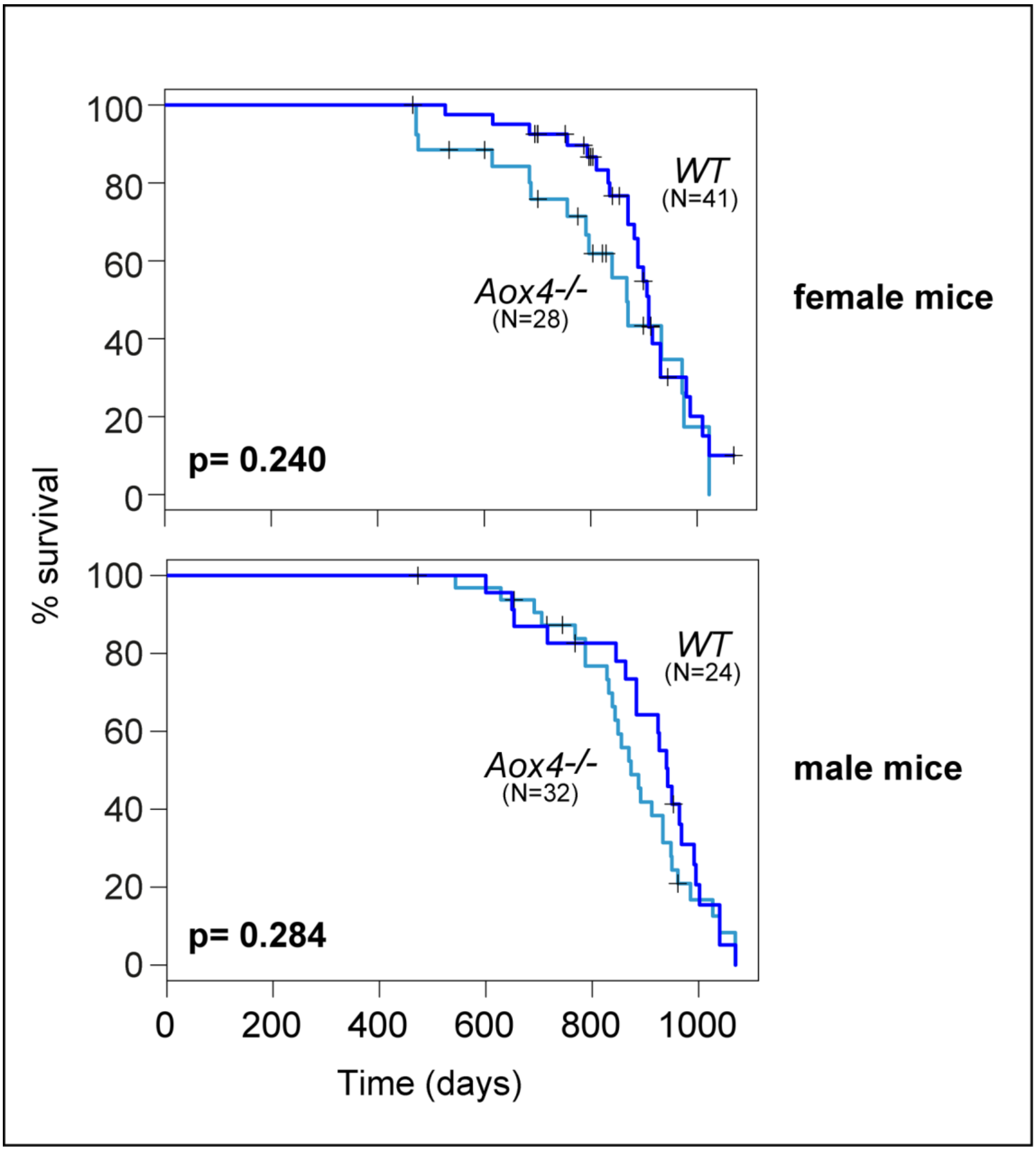

Suppl. Fig. S17
